# Amyloid β-protein oligomers promote the uptake of tau fibril seeds potentiating intracellular tau aggregation

**DOI:** 10.1186/s13195-019-0541-9

**Published:** 2019-10-18

**Authors:** Woo Shik Shin, Jing Di, Qin Cao, Binsen Li, Paul M. Seidler, Kevin A. Murray, Gal Bitan, Lin Jiang

**Affiliations:** 1 Department of Neurology, David Geffen School of Medicine, UCLA, 635 Charles E Young Drive South, Los Angeles, CA 90095 USA; 2 Departments of Chemistry and Biochemistry and Biological Chemistry, UCLA-DOE Institute, UCLA, Los Angeles, CA 90095-1570 USA; 3 Brain Research Institute, and Molecular Biology Institute, UCLA, Los Angeles, CA 90095 USA

**Keywords:** Amyloid beta, Tau, Biosensor cell, Oligomer, Alzheimer’s disease

## Abstract

**Background:**

Repeated failure of drug candidates targeting Alzheimer’s disease (AD) in clinical trials likely stems from a lack of understanding of the molecular mechanisms underlying AD pathogenesis. Recent research has highlighted synergistic interactions between aggregated amyloid-β (Aβ) and tau proteins in AD, but the molecular details of how these interactions drive AD pathology remain elusive and speculative.

**Methods:**

Here, we test the hypothesis that Aβ potentiates intracellular tau aggregation, and show that oligomeric Aβ specifically exacerbates proteopathic seeding by tau. Using tau-biosensor cells, we show that treatment with sub-toxic concentrations of Aβ oligomers, but not monomers or fibrils, “primes” cells, making them more susceptible to tau seeding. The treatment with Aβ oligomers enhances intracellular tau aggregation in a dose-dependent manner when the cells are seeded with either recombinant or brain-derived tau fibrils, whereas little or no aggregation is observed in the absence of Aβ-oligomer priming.

**Results:**

Priming by Aβ oligomers appears to be specific to tau, as α-synuclein seeding is unaffected by this treatment. Aβ oligomer-enhanced tau seeding also occurs in primary mouse neurons and human neuroblastoma cells. Using fluorescently labeled tau seeds, we find that treatment with Aβ oligomers significantly enhances the cellular uptake of tau seeds, whereas a known tau-uptake inhibitor blocks the effect of Aβ on tau uptake.

**Conclusion:**

The ability of Aβ to promote tau seeding suggests a specific and plausible mechanism by which extracellular Aβ initiates a deleterious cascade that is unique to AD. These data suggest that the Aβ-mediated potentiation of tau uptake into cells should also be taken into account when designing Aβ-targeted therapeutics.

## Background

Amyloid plaques and neurofibrillary tangles (NFTs), comprising Aβ and hyperphosphorylated tau, respectively, are the two major hallmarks of Alzheimer’s disease (AD) pathology [1–3]. The relationships among Aβ, tau, and neurodegeneration in AD are not fully understood [4, 5]. Although the amyloid cascade hypothesis has posited that Aβ aggregation is the initiating pathologic event in AD [6, 7], biomarker and pathology studies have shown a strong correlation between NFT accumulation, neurodegeneration, and clinical decline, whereas plaque pathology correlated poorly with AD progression [8–12]. Moreover, despite promising results in pre-clinical models, therapies that reduce plaque load have not yielded significant benefits in clinical trials for AD. The limited understanding of the link between Aβ accumulation and tau deposition in AD is a key piece that is missing from our knowledge of the disease mechanism, and may factor into failures of existing Aβ therapies in clinical trials.

The interplay between Aβ and tau is exemplified in animal models and biomarker studies of AD patients [13–16]. Studies in animal models show synergistic enhancement of tau accumulation in the presence of Aβ in the cortex of young mice overexpressing the frontotemporal-dementia-associated variant P301L-tau. In wild-type mice, Aβ plaques enhance tau seeding and pathology [17, 18]. Biomarker studies in patients concluded that the progression of AD dementia is driven by the synergistic interaction between Aβ and tau [19–22]. This body of work provides the basis for our hypothesis that Aβ and tau synergize to create a defining pathology in AD, the details of which are the focus of our current study.

Aβ and tau aggregate to form small soluble oligomers, and large insoluble fibrils that are seen in AD and related diseases [14, 23]. Several studies have shown that oligomeric and fibrillar species contribute differently to disease progression. For example, soluble Aβ oligomers are thought to be a major toxic agent in AD [24]. Tau oligomers and fibrils have been proposed to template the conversion of monomers into aggregates in recipient cells, leading to pathological spread in the brain [25–28]. Cell culture assays are useful tools for dissecting the contribution of specific assemblies in each pathological process. Experiments using primary neurons or neuronal cell lines have shown that application of Aβ oligomers increased tau phosphorylation, demonstrating a link between Aβ toxicity and tau pathology [29]. However, it is unclear how a direct interaction might occur between Aβ and tau in pathological tau seeding.

Here, we sought to explore how different Aβ assemblies might contribute to the process of tau seeding. We investigated the relationship between different assemblies of Aβ—freshly prepared, oligomeric, and fibrillar—and tau seeding using several cell-culture models, including FRET-based tau biosensor cells [30, 31], human neuroblastoma cells, and primary hippocampal neurons from transgenic mice expressing human P301S tau. We also explored the mechanism by which Aβ oligomers affect cellular uptake of tau seeds. Our findings connect toxic Aβ oligomers to tau seeding, a currently missing link in our understanding of AD pathology.

## Results

### Preparation of defined self-assembly states of Aβ and tau

**Fig. 1:**
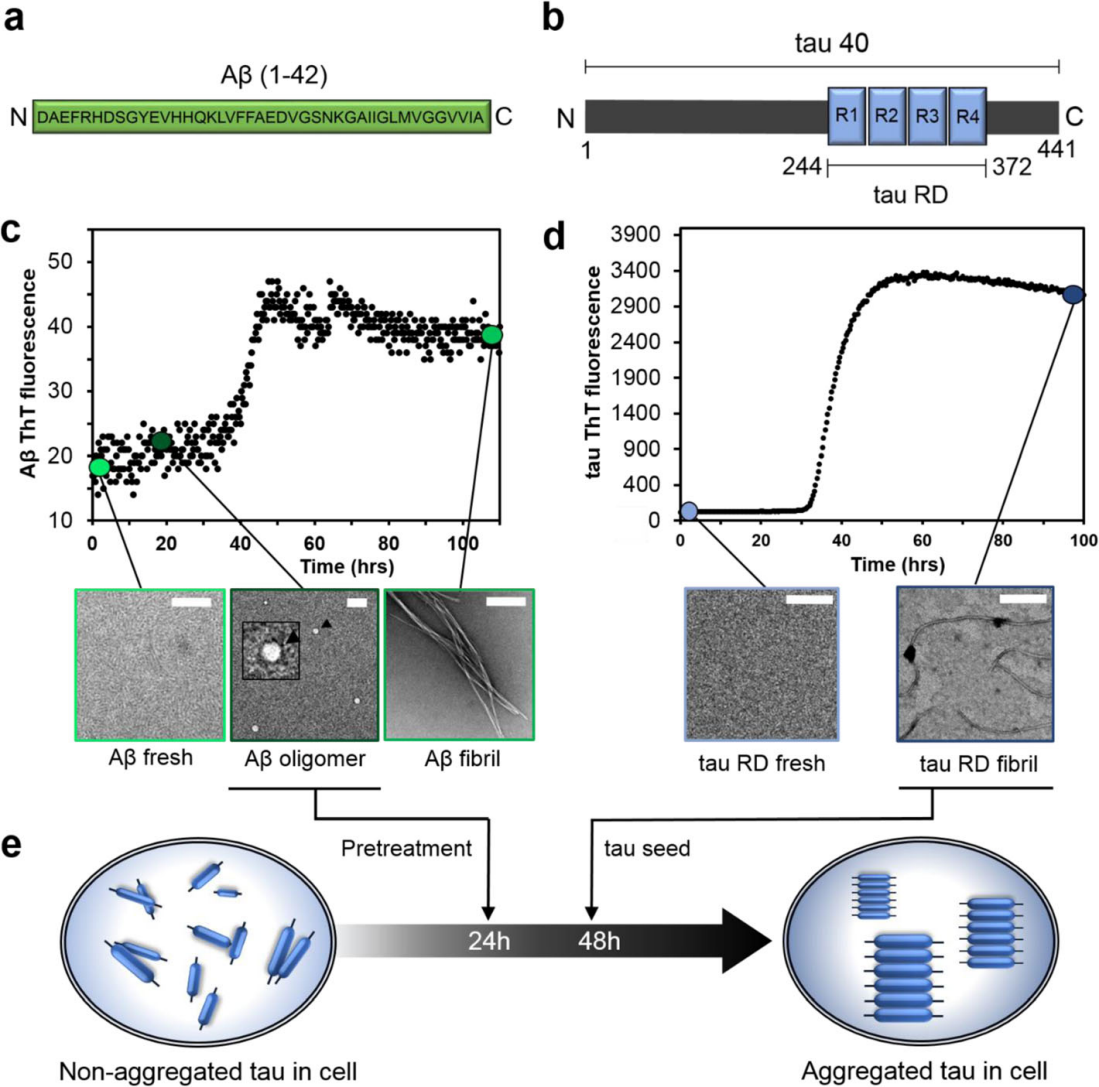
Characterization of Aβ and tau self-assembly. **a** The sequence of the Aβ(1–42). **b** A schematic of full-length tau (tau40, residue 1–441), which contains four tandem repeats (R1–R4) in the repeat domain (tau RD, residues 244–372). **c** ThT fluorescence (upper panel) and EM images (lower panel) of Aβ42 at different incubation times demonstrate that freshly prepared Aβ42 self-assembles and aggregates into spherical oligomers at 18 h and unbranched fibrils at 100 h. The inset highlights the spherical shape of the Aβ42 oligomers. The scales bar denotes 200 nm. **d** ThT fluorescence (upper panel) and EM images (lower panel) of tau RD. **e** A schematic representation of the timeline used for seeding experiment. Different Aβ42 self-assembly states and tau seeds were sequentially added to cells at 24 h and 48 h, respectively

To determine which forms of self-assembled or aggregated Aβ might promote tau seeding, we first expressed and purified recombinant Aβ and tau and prepared different self-assembled forms of each. Recombinant Aβ (1–42) (Fig. 1a) was expressed and purified as described previously [32]. To eliminate pre-formed aggregates during purification, the protein was fractionated using size-exclusion chromatography (SEC), yielding a major peak with an apparent retention time of a trimer (Additional file 1: Figure S1a), which previously has been shown to consist of a mixture of monomers and small oligomers, defined as low molecular weight (LMW) Aβ [33]. This LMW Aβ showed a major band when analyzed by native-PAGE at ~ 20 kDa and a smeary band consistent with molecular weights ranging from 70 to 200 kDa (Additional file 1: Figure S1b), likely representing the immediate self-assembly of Aβ in the PBS solution. Examining the preparation by electron microscopy (EM) showed occasional spherical structures, but mostly the species in freshly prepared Aβ were too small to be observed by EM (Additional file 1: Figure S1c). The freshly prepared Aβ was then quiescently incubated at 37 °C at 10 μM in PBS to generate different self-assembly states. We monitored the self-assembly of Aβ by thioflavin T (ThT) fluorescence (Fig. 1c). After incubation for 18 h, native PAGE showed a smeary band consistent with molecular weight ≥ 250 kDa, suggesting the presence of high molecular weight oligomers composed of ≥ 55 copies of the Aβ peptide (~ 4.5 kDa per monomer) (Additional file 1: Figure S1b). EM images of these samples, termed oligomeric Aβ, showed abundant quasi-spherical structures with diameters ranging between 20 and 60 nm (Additional file 1: Figure S1d,e). Finally, after ~ 100 h of incubation, Aβ formed typical, unbranched amyloid fibrils (Fig. 1c). The native PAGE lane for the fibril sample shows no bands (Additional file 1: Figure S1b), indicating a complete conversion of the samples into insoluble fibrils unable to migrate on the gel, which is also confirmed by the plateau in the ThT kinetics (Fig. 1c).

Recombinant tau repeat domain (RD, Fig. 1b) was used to generate fibrillar seeds in PBS containing 20 mM DTT at 37 °C. We did not use aggregation inducers, such as heparin, because of their potential effect on intracellular tau seeding/aggregation, complicating data interpretation. Under these conditions, following a lag phase of ~ 30 h, a sharp increase in ThT fluorescence was observed, suggesting tau fibril formation. In agreement with these results, EM images showed no aggregates at 0-h incubation and elongated fibrils after 100 h of incubation (Fig. 1d). In contrast to Aβ, for which quasi-spherical oligomers were observed at intermediate time points (from 10 to 30 h), we did not observe oligomers of tau RD by EM.

### Aβ oligomers promote tau aggregation seeded by tau RD, full-length tau, or brain extracts from a tauopathy mouse model

**Fig. 2:**
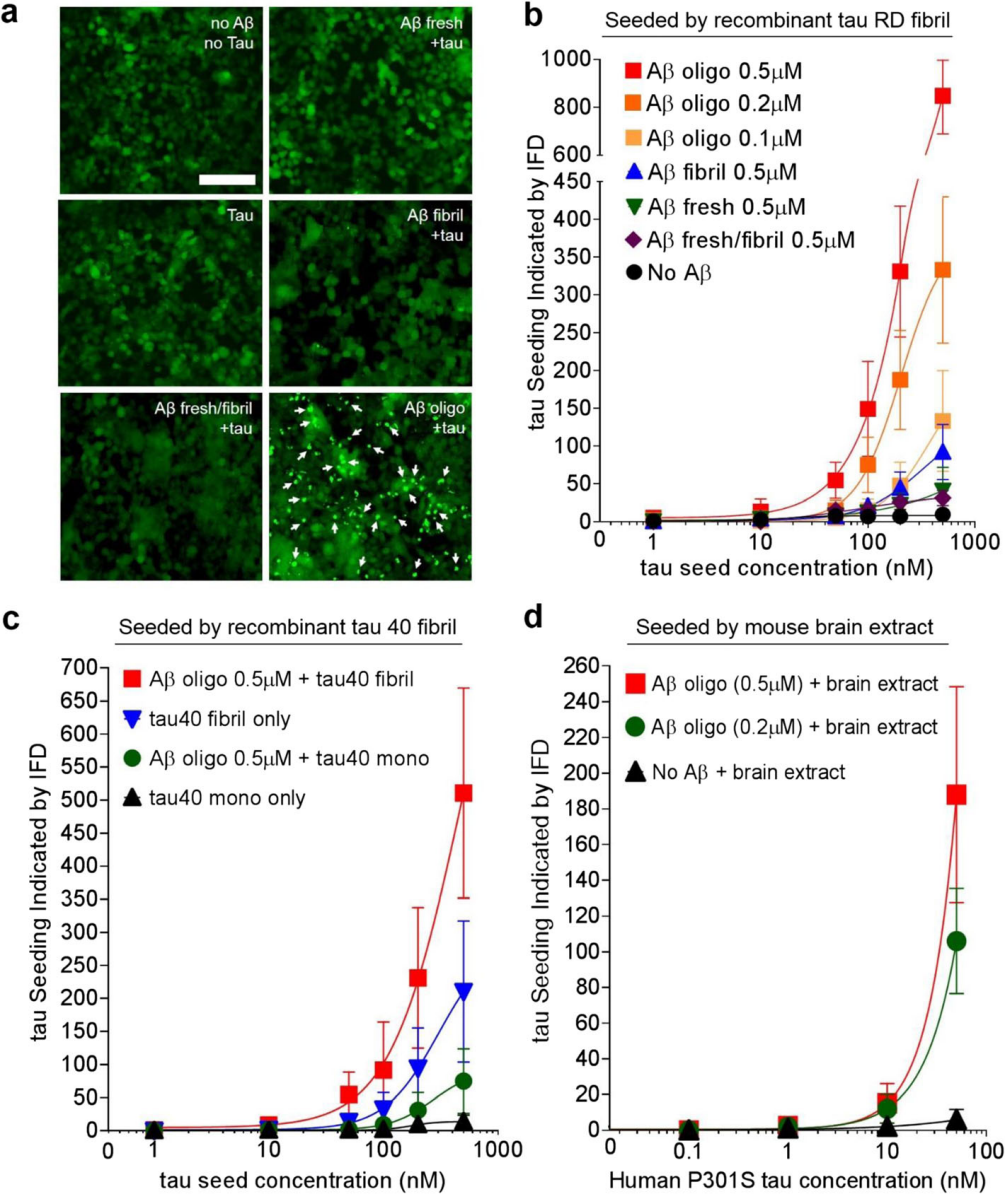
Pretreatment with Aβ oligomers but not Aβ freshly prepared or Aβ fibrils promotes tau seeding. **a** Fluorescence microscopy images of tau-biosensor cells treated with 500 nM of freshly prepared, oligomeric, or fibrillar Aβ followed 24-h later by 200 nM tau seeds. Tau aggregates are indicated by white arrows. The scale bar denotes 50 μm. **b**–**d** Flow-cytometry-based FRET quantification of intracellular tau aggregation seeded by recombinant tau RD fibrils (**b**), tau 40 fibrils (**c**), or PS19 mouse brain extracts (**d**). The enhancement by Aβ pretreatment is much stronger for Aβ oligomers (red), compared to Aβ fresh (green) and fibrils (blue), and occurs in a dose-dependent manner. **c** Aβ promotes intracellular tau aggregation seeded by full-length tau. **d** Aβ promotes seeding with brain extract from mice expressing human full-length P301S tau. ELISA was used to quantify human tau expressed in mouse brain extracts. The data are presented as mean ± SD (*n* = 6–9)

Following the preparation of different aggregated species of Aβ, we assessed their impact on tau seeding using the HEK293T tau biosensor cell line developed by Diamond and co-workers for monitoring and quantifying intracellular tau aggregation and seeding [34, 35]. Expressing tau RD containing the disease-associated P301S substitution fused to either CFP or YFP, the biosensor cells produce a FRET signal upon aggregation of tau, which can be quantified using flow cytometry. As a control, we first tested the effect of Aβ on the biosensor cells in the absence of tau seeds. The cells were treated with up to 200 nM freshly prepared, oligomeric, or fibrillar Aβ without the addition of transfection reagent, and FRET was quantified by flow cytometry. As expected, minimal tau aggregation (integrated FRET density (IFD): < 10, Additional file 1: Figure S2) was observed after 48 h, and even after 2 weeks (Additional file 1: Figure S3), demonstrating that neither form of Aβ induced tau aggregation without the addition of tau seeds in these cells. Our additional control experiments using tau RD seeds on their own, again not mixed with transfection reagents or any other additive, showed no measurable intracellular aggregation in the biosensor cells (Fig. 2a, b).

Next, to test whether Aβ facilitates tau seeding, biosensor cells were seeded with sonicated tau RD fibrils 24 h after adding freshly prepared, oligomeric, or fibrillar Aβ, and the resulting levels of intracellular tau aggregation were quantified after an additional 48-h incubation. Treating cells with 100–500 nM Aβ oligomers 24 h before addition of tau RD seeds led to a significant, dose-dependent increase in intracellular tau aggregation. The aggregates were visualized as bright puncta by fluorescence microscopy (Fig. 2a, white arrows) and were quantified by flow cytometry (Fig. 2b). Treatment of the cells with Aβ fibrils also promoted tau aggregation, yet to a substantially lower extent (~ 10%) than Aβ oligomers. Treatment with freshly prepared Aβ or a mixture of freshly prepared Aβ and Aβ fibrils did not promote tau aggregation.

We further asked whether Aβ oligomers could facilitate intracellular tau aggregation seeded not only by the tau RD, but also by full-length recombinant human tau (tau40) or by brain extracts from transgenic PS19 mice expressing human 1N4R tau containing the P301S substitution [36]. Addition of tau40 fibrils at 100–500 nM induced a low level of tau aggregation in the biosensor cells (Fig. 2c, blue curve). Pre-treatment of the cells with 500 nM Aβ oligomers doubled the seeding effect of tau40 fibrils (red curve). Following the same trend, pre-treatment with Aβ oligomer led to a small increase in seeding by freshly prepared tau40 (green and black curves). When we quantified tau aggregation seeded by PS19-mouse brain extracts, containing human 1N4R P301S tau, priming with 200 or 500 nM Aβ oligomers dose-dependently increased tau seeding by 15 and > 30 fold, respectively, compared to brain extract added without Aβ pre-treatment (Fig. 2d). Representative fluorescence microscopic images of these cells are shown in Additional file 1: Figure S4. Though tau RD, tau40, and PS19 brain extracts had different seeding capacities, in all cases, priming with Aβ oligomers substantially enhanced the seeding of tau.

We also assessed whether the priming of the biosensor cells by Aβ oligomers required a preincubation time, or could be achieved immediately. When tau seeds were added to the cells simultaneously with Aβ oligomers, we did not observe promotion of tau seeding (Additional file 1: Figure S5), suggesting that sufficient incubation time (~ 24 h) of the pretreatment was needed for Aβ oligomer to prime the cells for enhancing tau seeding.

### Aβ oligomers do not facilitate α-synuclein seeding

An important mechanistic question is whether Aβ oligomers promote seeding of tau specifically or could facilitate seeding of other amyloidogenic proteins in a non-specific manner. To address this question, we examined the effect of Aβ oligomers on seeding of α-synuclein, whose aggregation has been associated with Parkinson’s disease and other synucleinopathies [37]. Similar to tau, α-synuclein pathology has been reported to accumulate through brain regions, and α-synuclein aggregates often are found in the AD brain [38]. Using HEK293T biosensor cells co-expressing CFP- or YFP-fused A53T-α-synuclein, we added up to 1 μM sonicated α-synuclein fibrils in the absence or presence of Aβ-oligomer priming. In contrast to the experiments using tau seeds and tau-biosensor cells, we did not observe an enhancement in the seeding of α-synuclein in α-synuclein-biosensor cells pre-treated with up to 200 nM Aβ oligomers compared to cells that were not pre-treated (Additional file 1: Figure S6), suggesting that the seeding enhancement by Aβ oligomers is specific to tau.

### Aβ oligomers facilitate tau seeding in primary neurons and human neuroblastoma cells

**Fig. 3:**
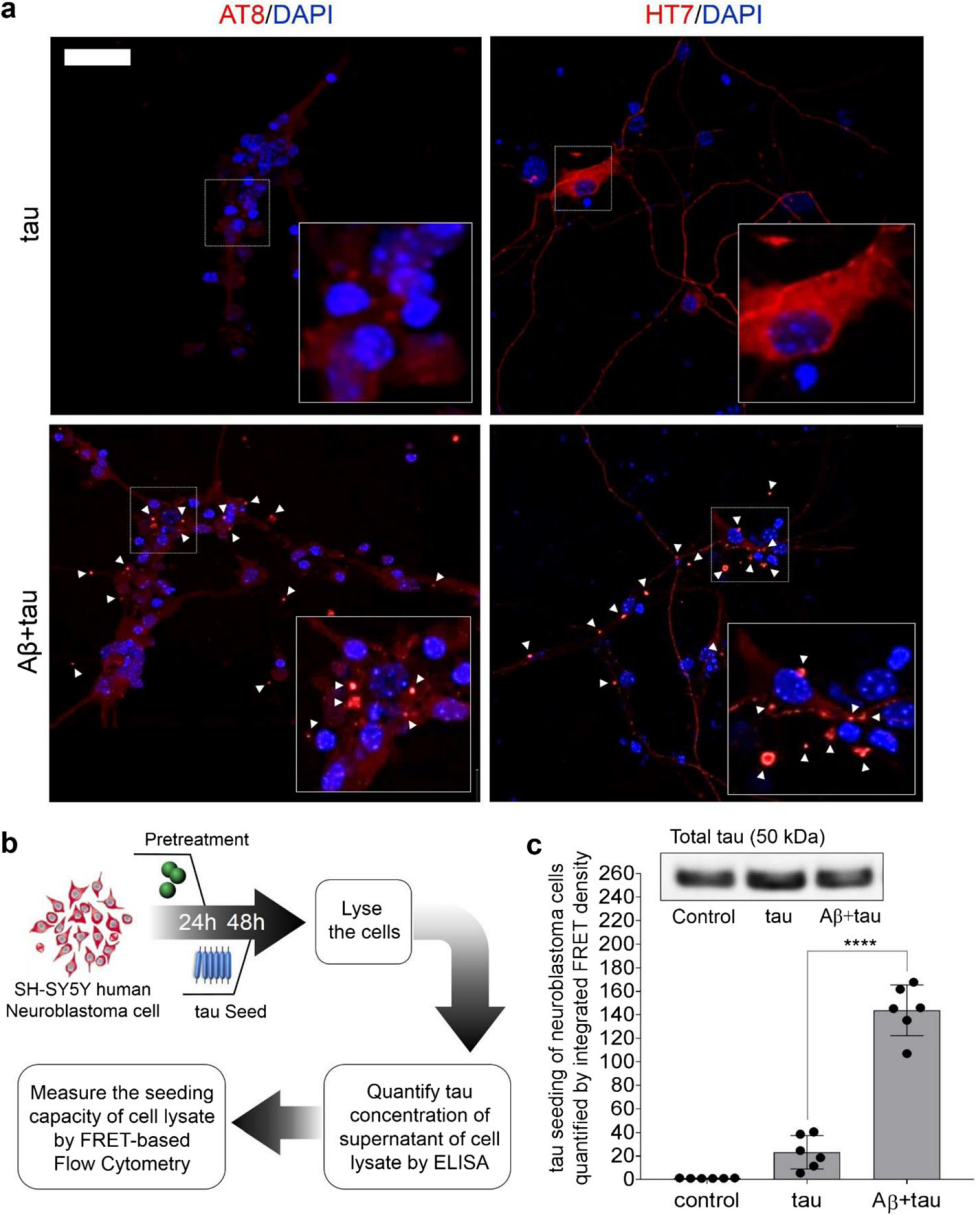
Aβ oligomers promote tau seeding in primary hippocampal neurons and human neuroblastoma cells. **a** 500 nM tau seeds were added to primary hippocampal neurons from PS19 mice with (top panels) or without (bottom panels) 24 h pretreatment of 200 nM Aβ oligomers. Immunostaining with tau antibodies HT7 (left) or AT8 (right) was performed 24 h after the addition of tau seeds. Nuclei were stained with DAPI (blue). The scale bar denotes 20 μm. Tau inclusions are highlighted by white arrows. **b** A schematic representation of the assays used to measure Aβ promotion of tau seeding in neuroblastoma SH-SY5Y cells. Cells were treated with Aβ oligomers at 24 h and tau seeds at 48 h and then lysed at 72 h. ELISA was used to measure tau concentration in the lysates, then seeding was measured in the tau-biosensor cells, and the seeding capacity of the supernatant was measured by FRET-based flow cytometry. **c** Quantification of tau seeding in SH-SY5Y cells with or without pretreatment with Aβ oligomers. The representative western blot above the bar graph shows that a similar amount of total tau in the SH-SY5Y cell lysates was used in each case. Data are mean ± SD (*n* = 6, ****p < 0.0001, one-way ANOVA)

The FRET-based biosensor cells are a convenient tool for sensitive quantification of tau or α-synuclein seeding, but may not fully represent seeding in neurons. Therefore, we sought to determine whether Aβ oligomers facilitate tau seeding in primary mouse neurons and a human neuroblastoma cell line. Primary hippocampal neurons were isolated from the PS19 mice and treated with tau seeds with or without Aβ-oligomer priming. Because this cellular system does not express fluorescently labeled tau and is not applicable to FRET-based flow cytometry, we used confocal microscopy instead to qualitatively assess seeding (Fig. 3a). When immunostained with antibodies HT7 or AT8, which recognize total human tau and phosphorylated tau (at Ser 202 and Thr 205), respectively [39], primary hippocampal neurons treated with 500 nM tau RD seeds alone showed a few bright fluorescent puncta, while untreated cells showed no such puncta. Treatment with 200 nM Aβ oligomers 24 h before the addition of tau seeds led to a marked increase in red fluorescent puncta (indicated by white arrows, Fig. 3a, bottom panels).

To quantitatively measure tau seeding in neuronal cells, we established a cellular assay in which human SH-SY5Y neuroblastoma cells were incubated with tau seeds with or without pre-treatment with Aβ oligomers. The cells were washed extensively with PBS to remove any tau and Aβ that might have been present in the culture media, lysed, and their lysates centrifuged to remove insoluble material and cell debris. The clarified cell lysates (tau concentration of 26 nM, measured by Western blot and ELISA, Additional file 1: Figures S7-S8) were added to the tau biosensor cells for measurement of seeding (Fig. 3b). Priming the SH-SY5Y cells with Aβ oligomers yielded > 4-fold increase in tau aggregation in biosensor cells compared to the SH-SY5Y cells without Aβ-oligomer priming (Fig. 3c).

### Aβ oligomers enhance tau seeding by promoting seed internalization

**Fig. 4:**
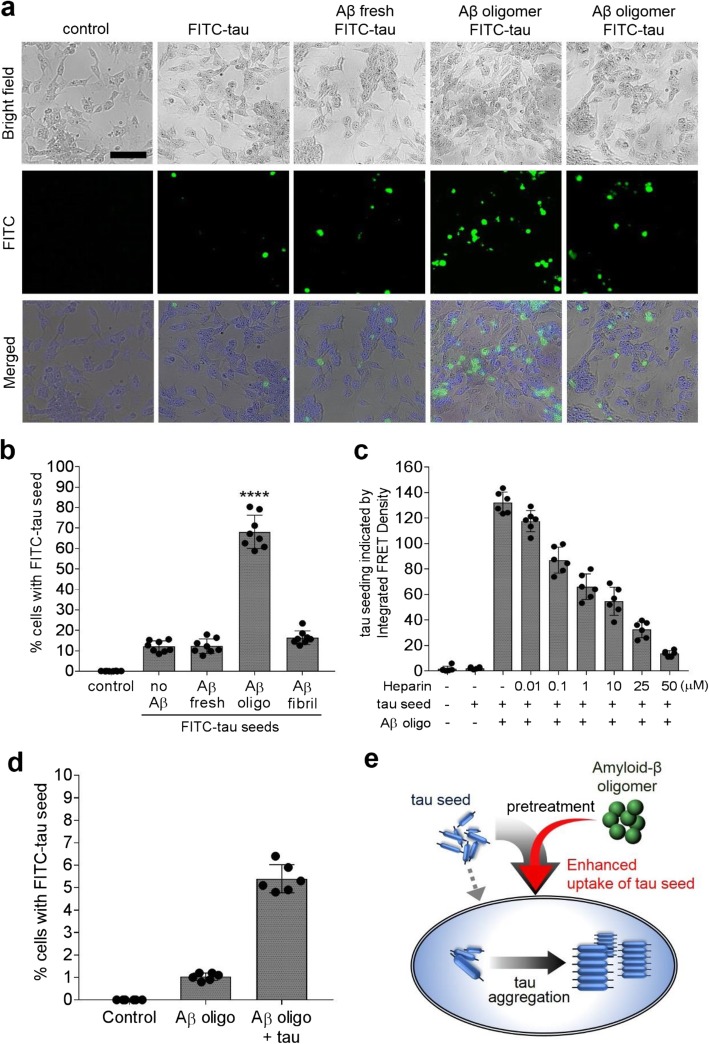
Aβ oligomers potentiate intracellular tau aggregation by promoting tau seed uptake. **a**, **b** Fluorescence-microscopy images (**a**) and flow cytometry-based quantification (**b**) of internalized fluorescein-labeled tau seed (500 nM, green) in SH-SY5Y cells with or without pretreatment with 200 nM freshly prepared, oligomeric, or fibrillar Aβ. Untreated SH-SY5Y cells were used as a negative control. After extensive PBS washing, cells were fluorescently imaged and subsequently harvested with trypsin treatment for flow cytometry. The data are presented as mean ± SD (*n* = 8, ****p < 0.0001, one-way ANOVA). The scale bar denotes 20 μm. **c** Various concentrations of heparin, from 10 nM to 50 μM, were applied to HEK293T tau biosensor cells 1 h before Aβ pretreatment, and tau seeding was quantified as integrated FRET density using flow cytometry. **d** The same trend of Aβ-oligomer mediated enhancement of labeled tau seed internalization in HEK293T cells. Heparin was added 1 h before Aβ pretreatment. The data are presented as mean ± SD (*n* = 6). **e** A diagram of Aβ-promoted tau seeding

To visualize the cellular uptake of tau seeds, we conjugated fluorescein to tau RD (FITC-tau), allowed the protein to form fibrils, and prepared seeds. We then primed SH-SY5Y cells with 200 nM freshly prepared, oligomeric, or fibrillar Aβ for 24 h, followed by seeding with 500 nM FITC-tau fibrils. After extensive washing with PBS, the cells were inspected by fluorescence microscopy (Fig. 4a) and seeding was quantified using flow cytometry (Fig. 4b). Without Aβ-oligomer priming, 12 ± 3% of the SH-SY5Y cells showed internalized FITC-tau seeds. Treatment with freshly prepared Aβ (12 ± 4%) or Aβ fibrils (16 ± 3%) did not affect FITC-tau internalization. Priming with oligomeric Aβ increased the uptake of FITC-tau seeds significantly to 64 ± 8%, suggesting that Aβ oligomers facilitated tau seeding by promoting the internalization of the seeds. Aβ-promoted internalization of tau seeds was also observed in wild-type mouse primary neurons (Additional file 1: Figure S9).

Lastly, to investigate whether inhibition of tau seed uptake could block Aβ promoted tau seeding, we tested heparin, a known tau uptake inhibitor [40], in the tau biosensor cells. Flow-cytometry analysis revealed that increasing concentrations of heparin, 1 to 50 μM, decreased the Aβ-promoted tau seeding in a dose-dependent manner (Fig. 4c). We confirmed that heparin inhibits tau-seed uptake using wild-type HEK293T cells instead of the biosensor cells because of spectral overlap between the fluorescence of FITC and YFP/CFP. Similar to the SH-SY5Y cells, Aβ oligomers increased the internalization of FITC-tau seeds in wild-type HEK293T cells > 5-fold compared to untreated cells (Fig. 4d). Addition of 10 μM heparin reduced tau internalization to the level of non-primed cells.

## Discussion

Studies in rodents and on clinical specimen have demonstrated a synergistic interaction between Aβ and tau in the development of AD [13–15], but the mechanistic details of this synergy have yet to be fully elucidated. In this study, we focused on tau seeding, which is correlated to AD progression, and investigated how Aβ affects this seeding process. Using biochemical and cellular assays, we aimed here to dissect which self-assembly states of Aβ contribute to tau seeding and aggregation. We found that tau seeding is greatly enhanced by Aβ oligomers, but not by freshly prepared Aβ or Aβ fibrils. The enhancement of seeding was not limited to a particular construct of tau and was apparent for seeds that were prepared from tau RD, full-length tau, and mouse brain extract containing human P301S tau. In addition, seeding enhancement was not restricted to a particular cell type and was observed in multiple cellular systems including HEK293T cells, human neuroblastoma cells, and mouse primary hippocampal neurons. Our data illustrate that 100–200 nM of Aβ oligomers effectively facilitate tau seeding (Additional file 1: Figures S10 and S11), which are below the micromolar concentrations of Aβ oligomers typically used in cellular toxicity studies [41, 42]. Notably, these concentrations are in the same range as the reported physiological Aβ concentration, between 42 nM and 195 nM in AD patients [43, 44].

Previous studies have proposed that soluble Aβ oligomers are the primary effector of neurotoxicity in AD by inducing cellular stress through non-specific mechanisms, including oxidative stress and pore formation [45, 46]. At micromolar concentrations, our Aβ-oligomer preparation with similar morphology to previous studies [41, 42] also exhibited cytotoxicity to multiple cell types, including primary neurons [47]. We demonstrate here that at physiologically relevant concentrations, Aβ oligomers exhibit a heretofore unknown deleterious activity—facilitation of tau seeding, providing a plausible mechanism that links Aβ to the stimulation of tau pathology, as is the case in AD. Moreover, the data provides a possible explanation to the sharp difference between the high prevalence of AD in the aging population as opposed to other tauopathies, which are much rarer. Aβ oligomers are unique to AD and are not known to be part of the pathological mechanism in rare tauopathies. Thus, we propose that AD is not just a coincidental combination of two unrelated toxic proteoforms, aggregated Aβ and tau, but rather is the manifestation of a synergistic exacerbation of tau spreading by Aβ oligomers. In agreement with this idea, a recent study demonstrated that suppression of tau gene expression was substantially less effective at rescuing neuronal impairment in transgenic mice expressing both human Aβ and tau compared to mice expressing tau alone [48].

Our data demonstrate that the Aβ-oligomer promotes seeding that is apparently unique to tau protein. α-Synuclein seeding, which is a similar process to that of tau, is not facilitated by Aβ oligomers. The specific activation of tau seeding by Aβ oligomers may explain not only the higher prevalence but also mechanistic aspects of AD compared to other tauopathies, such as primary age-related tauopathies (PART), the NFTs of which are indistinguishable from those of AD [49]. PART, which does not involve any Aβ pathology, has characteristic NFT pathology localized to the medial temporal lobe with low levels of tau spreading to other brain regions [50]. In comparison, AD, which involves both Aβ and tau, is characterized by the accumulation of NFT pathology in broader brain regions. These different capacities of NFT accumulation between AD and PART may reflect differences in the ability of tau to spread in AD by Aβ oligomers, whereas in the absence of Aβ oligomers, the spread of tau is slower and more limited to specific brain regions, as in the case of PART.

Aβ oligomers could promote tau seeding by several mechanisms. One possible mechanism is the formation of Aβ channels/pores in the cell membrane through which tau seeds would enter. Multiple reports have suggested that Aβ oligomers could exert neurotoxicity by forming pores or channel-like structures in the plasma membrane [41, 51, 52]. Formation of Aβ pores likely occurs over a short period of time (1 to 2 h) [53]. Our experiments suggest that sufficient incubation time with Aβ oligomers is needed for enhancement of tau seeding as pretreatment times of 0, 1, and 3 h also show no enhancement of tau seeding (Additional file 1: Figure S5). In addition, if tau seeding was facilitated by Aβ-oligomer pore formation in the plasma membrane, enhancement of α-synuclein seeding would be expected, but Aβ pretreatment of α-synuclein biosensor cells show no enhancement of α-synuclein seeding (Additional file 1: Figure S6). In short, our studies suggest the observed promotion by Aβ oligomers is not likely to be directly related to pore formation.

Another possible mechanism is cross-seeding of intracellular tau aggregation by Aβ oligomers, as proposed previously [54]. However, none of the Aβ self-assemblies induced tau aggregation when tau seeds were not added 24 h later, and thus, it is unlikely that Aβ cross-seeded tau aggregation in our assays. Taken together, our data show that the Aβ-mediated increase in tau seeding is a specific process that neither involves cross-seeding between Aβ and tau nor the formation of pores in the plasma membrane by Aβ oligomers, but rather that Aβ oligomers prime the cells for tau internalization by some other currently unknown mechanism.

Using our cellular assays, we demonstrated that pretreatment with sub-toxic concentrations of Aβ oligomers significantly enhances the cellular uptake of tau seeds, leading to a substantial increase of intracellular tau aggregation. This potentiation is inhibited by the addition of heparin, a tau uptake inhibitor (Fig. 4e). We reach this finding by using more physiologically relevant cellular assays of tau seeding, compared to previous cellular studies which typically have used transfection agents, such as lipofectamine, to achieve efficient transduction of tau seeds into cells [13]. Using such transfection agents allows for sensitive measurements of seeding, but does not reflect tau seeding as it would occur in vivo. In contrast, artificial transfection reagents were not utilized in our study, enabling us to discover the seeding-enhancing capability of Aβ oligomers in a more physiologically relevant way. In future studies, in addition to revealing a potentially relevant heretofore unknown mechanism that may underlie the propagation of tau pathology in AD, the cellular assays we developed should be suitable for high-throughput compound screening for drugs targeting Aβ-enhanced tau seeding. New drug design and development based on blocking Aβ-promoted tau seeding by inhibiting Aβ-oligomer–cell interactions [55], preventing tau seed uptake, or a combination of both could present new opportunities with greater promise to delay or even halt AD progression.

## Conclusion

In our study, we demonstrated how different species of Aβ self-assemblies (oligomers, fibrils, etc.) might contribute to the process of tau aggregation. We investigated that the ability of oligomeric Aβ to promote tau seeding suggests a specific and plausible mechanism by which extracellular Aβ initiates a deleterious cascade that is unique to AD using several cell-culture models, including FRET-based tau biosensor cells, human neuroblastoma cells, and primary hippocampal neurons from transgenic mice expressing human P301S tau. We also explored the mechanism by which Aβ oligomers affect cellular uptake of tau seeds. Our data connect toxic Aβ oligomers to tau seeding, a currently missing link in understanding of AD pathology and suggest that the Aβ-mediated potentiation of tau uptake into cells should also be taken into account when designing Aβ-targeted therapeutics.

## Materials and methods

### Animals

Animal care was conducted in compliance with the US Public Health Service Guide for the Care and Use of Laboratory Animals, and the procedures were approved by the Institutional Animal Care and Use Committee at the University of California, Los Angeles. Eight-month-old P301S (PS19) transgenic mice on (C57BL/6 x C3H) F1 background and wild-type mice were used for the study.

### Aβ (1–42) expression and purification

Aβ (1–42) was expressed and purified as described previously [32]. Briefly, *Escherichia coli* BL21 plyS (DE3) cells were transformed with a plasmid encoding Aβ (1–42) conjugated to maltose binding protein (pET28a-MBP-Aβ42). The expression of the fusion protein MBP-Aβ42 was induced with 1 mM of isopropyl-β-D-thiogalactopyranoside (IPTG) at 30 °C for 4 h. The culture was harvested and washed. The denatured fusion protein was purified using a HisTrap HP column (GE healthcare). After overnight dialysis, the fusion protein was cleaved overnight by TEV protease. The uncleaved protein and TEV were removed using Ni^2+^ affinity chromatography. The Aβ (1–42) in the flow-through was purified by RP-HPLC and lyophilized. To ensure the homogeneity of the Aβ sample, the lyophilized powder was dissolved in 60 mM NaOH and fractionated by SEC (GE, Superdex 200 Increase) in 20 mM NaOH to eliminate pre-formed Aβ aggregates. The final concentration was determined by a BCA assay, and the stock was stored in − 80 °C.

### Tau expression and purification

Human tau RD (residues 244–372) and full-length tau (tau40, residues 1–441) were expressed and purified as described previously [56]. Briefly, for the expression of tau RD, BL21 (DE3 GOLD) competent cells and a pNG2 vector was used. Protein expression was induced by addition of 0.5 mM IPTG for 3 h at 37 °C, and the cells were lysed by sonication. Cell lysates were then boiled for 20 min and centrifuged to remove all insoluble proteins. The remaining soluble protein was purified using a HighTrap SP ion-exchange column (GE Healthcare), and fractions were analyzed by SDS-PAGE/Coomassie blue staining. Enriched fractions were further purified using a HiLoad 16/600 Superdex 75 SEC column (GE Healthcare). Purified tau was concentrated to 50–100 mg/ml and stored at − 80 °C.

For the expression of human tau40, a C-terminal His-tag was used. After IPTG induction, the cells were harvested and collected. The protein was purified using a HisTrap HP column and then purified by ion-exchange chromatography and SEC as described for tau RD.

### Thioflavin T (ThT) kinetics of amyloid aggregation of Aβ and tau

Before the ThT kinetics assay, freshly SEC-fractionated Aβ was mixed with 60 mM HCl to neutralize the NaOH in the stock solution. After 3-min sonication, the Aβ sample was diluted in PBS to a final concentration of 10 μM and 30 μM ThT (CalbioChem) was added. The reaction mixture was filtered through a 0.2-μm filter, split into 3–4 replicates, and immediately placed in a Corning 96-well Nonbinding plate (black, nonbinding surface microplate). The ThT fluorescence signal was measured every 5 min in quiescent conditions using a Fluostar Omega plate reader (BMG Labtech, Offenburg, Germany) with excitation and emission wavelengths of 440 and 490 nm, respectively, at 37 °C.

Freshly purified tau RD and tau40 were diluted in PBS containing 2 mM DTT, 40 μM ThT to a final concentration of 250 μM in the absence of any aggregation inducers (i.e., heparin). The reaction mixture was split into 3–4 replicates and placed in 96-well plates (Corning 3881). ThT fluorescence intensity was measured every 15 min with double orbital shaking at 37 °C in the same plate reader until a plateau was reached.

### Measurement of tau seeding using HEK293T biosensor cells

Tau RD P301S FRET Biosensor (ATCC CRL-3275) cells were cultured and analyzed as described previously [31]. The cells were grown in DMEM (Dulbecco’s modifications of Eagle’s medium with l-glutamine and 4.5 g/l glucose) supplemented with fetal bovine serum (FBS), 100 units/ml of penicillin G, and 0.1 mg/ml of streptomycin sulfate, in a humidified atmosphere of a 5% CO_2_ at 37 °C. Trypsin-treated HEK293T cells were plated on collagen-coated flat 96-well plates at a density of 2.5 × 10^4^ cells/well in 200-μl culture medium and incubated at 37 °C in 5% CO_2_. After 24 h, the cells were treated with different self-assembly states of Aβ or with media alone. Tau fibrils (tau RD, tau 40 and mouse brain extract) were diluted with Opti-MEM (GIBCO) and sonicated for 10 min in an ultrasonic water bath. Twenty-four hours after the treatment with Aβ, the culture media were replaced with fresh media containing the tau seeds. Tau aggregation in the biosensor cells was visualized at 72 h by fluorescence microscopy using green channel (ex: 485; em: 520). At 96 h, the cells were harvested after extensive washing and treatment with trypsin. The harvested cells were prepared in 200 μl chilled buffer (HBSS, 1% FBS, 1 mM EDTA) and then stored at 4 °C until they were analyzed by FRET-based flow cytometry.

### Measurement of α-synuclein seeding using HEK293T biosensor cells

α-synuclein biosensor cells (a gift from Marc Diamond, UTSW) [30] were used for the study. Cells were grown in same DMEM growth media and 2.5 × 10^4^ cells were plated on collagen-coated flat 96-well plates. After 24 h, the cells were treated with 200 nM Aβ oligomers followed by addition of sonicated α-synuclein fibrils at 48 h. The preparation of recombinant α-synuclein fibrils was performed as described previously [57]. α-Synuclein seeding was visualized at 72 h by florescence microscopy, and at 96 h, the cells were harvested and prepared for FRET-based flow cytometry.

### Flow cytometry and data analysis of tau and α-synuclein seeding

Intracellular protein aggregation of tau or α-synuclein was quantified by FRET-based flow cytometry. The protocol was adapted from reference [31]. All experiments were performed using a Digital Analyzers LSRII (IMED) flow cytometer. The fluorescence intensities of the FRET pair (ex: 405 nm; em: 525/50 nm), CFP-fusion proteins (ex: 405 nm; em: 450/50 nm), and YFP-fusion proteins (ex: 488 nm; em: 525/50 nm) alone were measured. For each experiment, FRET signals of 20,000 cells per replicate were analyzed to differentiate the aggregated protein from the non-aggregated protein. FRET gating was introduced to exclude all of the FRET-negative cells and include the FRET-positive cells. Integrated FRET density (IFD), defined as the percentage of FRET-positive cells multiplied by the median fluorescence intensity of FRET-positive cells, was calculated for all analyses. All experimental data were analyzed using GraphPad Prism 7.0. Plots were fitted to a non-linear sigmoidal curve. The flow-cytometry quantification of protein aggregation was conducted for a minimum of three independent experiments with at least three replicates in each experimental condition.

### Measurement of tau seeding in human neuroblastoma cells

Human neuroblastoma SH-SY5Y cells were cultured in Iscove’s modified Dulbecco’s medium supplemented with 15% fetal bovine serum (FBS), 1% l-glutamine, and 1% penicillin/streptomycin. Cells were transferred to collagen-coated flat 6-well plates at a density of 3.0 × 10^6^ cells/well in 1-ml culture medium and incubated at 37 °C in 5% CO_2_. The cells were treated with Aβ oligomers at 24 h, followed by tau seeds at 48 h. At 72 h, the cells were lysed by sonication in 15% RIPA buffer and the lysate supernatants were harvested. ELISA was used for quantifying total tau concentration in the lysate supernatants. After normalization of tau concentration, the lysate supernatant was added to tau biosensor cells to measure its seeding capacity. After 48 h, the HEK293T cells were collected, and intracellular tau aggregation was quantified using FRET-based flow cytometry.

### Preparation of fluorescently labeled tau (FITC-tau) fibrils

Freshly purified tau protein (50 μM in PBS containing 20 mM DTT) was incubated with shaking at 37 °C for > 100 h until most of the protein was converted into fibrils (a plateau of ThT fluorescent signal monitoring tau aggregation was reached). The tau fibrils were then diluted in a reaction buffer containing 5 μM DTT, 10 mM HEPES, pH 7.4, and 100 mM NaCl to a final concentration of 8 μM. 0.025 mg of Alexa Fluor 488 NHS Ester per 200 μL was added into the reaction mixture and incubated for 1 h at room temperature and then overnight at 4 °C with end-over-end rotation. To stop the reaction, the unconjugated dye was quenched with 100 mM glycine in PBS for 1 h. The labeled fibrils were washed extensively with PBS buffer by filtration using a molecular weight cut-off of 10 kDa.

### Cellular uptake assay of tau fibril seeds

SH-SY5Y or HEK293T cells were used to measure cellular uptake of tau fibril seeds. 3 × 10^4^ cells were plated in collagen-coated, flat 96-well plates, incubated for 24 h, and then treated with freshly prepared Aβ, Aβ oligomers, or Aβ fibrils. Fluorescently labeled FITC-tau was diluted in Opti-MEM (GIBCO) and added to the cells after an additional 24-h incubation. Tau uptake was monitored using florescence microscopy (ex: 485 em: 520) 24 h later. After an additional 24-h incubation, cells were harvested after extensive washing with 1× PBS and flow cytometry was used to quantify the cellular uptake of tau fibril seeds by calculating the percentage of FITC-positive cells.

### Electron microscopy (EM)

Five-microliter aliquots from aggregation reactions were taken at different time points and applied to carbon-coated 400-mesh Formvar grids (Electron Microscopy Science), which had been glow-discharged using a Pelco Easy-Glow unit for 2 min immediately before applying the samples. The samples were wicked off after 1 min, stained with 2% uranyl acetate for 2 min, and analyzed using a JEOL JEM1200-EX transmission electron microscope.

### SDS-PAGE/Western blot

Cells were lysed by sonication in mild (15%) RIPA buffer, and the lysate supernatants were harvested by centrifugation for 20 min at 14,000 rpm in 4 °C. The collected supernatants were dissolved in LDS sample buffer (Thermo Fisher Scientific) and boiled for 5 min prior to loading on NuPAGE 4–12% Bis-Tris polyacrylamide gels. Following fractionation by SDS–PAGE, proteins were transferred to polyvinylidene difluoride membranes and blocked with 5% non-fat dry milk in Tris-buffered saline containing 0.1% Tween 20 (TBST) for 60 min at room temperature. The membranes were incubated for 2 h at room temperature with anti-human tau monoclonal antibody HT7 (Cell Signaling Technology, Danvers, MA) at a 1:1000 dilution. After 3-min washes with TBST, membranes were incubated with HRP-conjugated [donkey] anti-mouse secondary Anti-thymocyte globulin (Zymed, San Francisco, CA) at a 1:5000 dilution and washed three times again with TBST. The transferred proteins were visualized using an enhanced chemiluminescence (ECL) detection kit (HyGLO Quick Spray Kit, Denville Scientific).

### ELISA

Tau concentration was quantified using an anti-tau ELISA kit (Invitrogen, cat# KHB0041) according to the manufacturer’s instructions. After cell lysis as described above, the resulting supernatant was collected for analysis. Fifty microliters of each sample was analyzed in triplicates.

### Primary-neuron culture

Twelve-millimeter coverslips were placed into the wells of 24-well plates and coated with 500 μl 0.5 mg/ml poly-ornithine dissolved in 50 mM sodium tetraborate, pH 8.3, overnight at 4 °C. After rinsing with PBS, pH 7.4, the coverslips were coated with 5 μg/ml laminin dissolved in PBS for 2–3 h at 37 °C and then rinsed and stored in PBS at 4 °C. Pyramidal hippocampal neurons were isolated and cultured from P0 or P1 postnatal P301S mice (PS19 transgenic mouse line, Jackson Laboratories) as described previously [58]. Briefly, the hippocampi were dissected under a microscope and collected into a 15-ml tube using ice-cold PGB buffer containing 0.2 g BSA, 0.9 g glucose in 200 ml PBS, pH 7.4. The PGB buffer was replaced with a solution of 10 ml, 0.5 mg/ml papain, and 0.6 μg/ml DNAase (Sigma) in PGB, and the tissue was incubated for 20 min at 37 °C, rinsed twice with PGB buffer, and dissociated in 2 ml PGB buffer by trituration using a flame-polished, plugged glass pipette. Then, 8 ml of PGB buffer was added and the preparation was centrifuged at 400×*g* for 10 min. The supernatants were discarded, and 2 mL of complete medium (0.5 mM glutamine, 100× penicillin/streptomycin, 50× B-27 in neurobasal medium, Gibco) was added. The cell pellet was dissociated gently using a flame-polished glass pipette, and the cells were counted using a hemocytometer. Seventy thousand to 100,000 cells per well were plated in 0.5 mL of the complete medium on the previously prepared coverslips. The cells were maintained at 37 °C in a 5% CO_2_ atmosphere. Half of the medium was changed 1–2 times a week.

### Tau seeding and immunocytochemistry

On day 8 of the primary neuron culture, 200 nM Aβ oligomers in complete medium were added. Twenty-four hours later, 500 nM sonicated tau RD fibrils were added. After an additional 24-h incubation, the cells were fixed in 4% (v/v) paraformaldehyde in PBS for 15 min, washed three times in PBS, and permeated for 1 h at room temperature in blocking buffer containing 0.1% BSA, 5% donkey serum, 0.3% Tween-20 in PBS, pH 7.4. The blocking buffer then was removed, and the cells were stained with HT7 or anti-phosphorylated tau monoclonal antibody AT8 (ThermoFisher) at 1:1000 dilution in blocking buffer overnight at 4 °C, followed by Alexa-Fluor-555-conjugated donkey anti-mouse secondary antibody (ThermoFisher Scientific) at 1:500 dilution in blocking buffer for 1 h at room temperature. After washing in PBS, the coverslips were mounted on microscope slides using Prolong Gold Antifade reagent with DAPI (Thermo Fisher Scientific) and imaged using a Leica SP8-SMD Confocal Laser Scanning Microscopy Platform.

### Preparation of mouse brain extracts

Eight-month-old PS19 mice and age-matched WT mice were deeply anesthetized with isoflurane (0.5–1.5 vol% in oxygen) and sacrificed by decapitation. Brains were dissected and suspended in 10% (w/v) ice-cold TBS containing protease and phosphatase inhibitors cocktail (ThermoFisher). The tissue was homogenized at 4 °C using a probe sonicator (Omni Sonic Ruptor 250) at 30% power using 25 pulses. Lysates were centrifuged at 21,000×*g* for 15 min to remove cell debris. Supernatants were aliquoted and stored at − 80 °C.

### Statistical analyses

Statistical analyses for two-group comparison were performed by *t* test calculator (QuickCalcs, GraphPad) with the choice of unpaired *t* test. For multiple comparison, ordinary one-way ANOVA multiple comparisons were performed using GraphPad Prism software ver. 7.0 (San Diego, CA). All graphs were generated using Prism software.

## Supplementary information

**Additional file 1:** **Figure S1.** Characterization of Aβ (1–42) preparations. **Figure S2.** The effect of different Aβ species on tau aggregation in the absence of tau seeds in biosensor cells. **Figure S3.** When treated Aβ oligomers up to two weeks in the absent of tau seeds, biosensor cells display negligible tau aggregation. **Figure S4.** Aβ oligomer pretreatment promotes intracellular tau aggregation when biosensor cells are seeded with brain extracts from transgenic mice expressing human P301S-tau. **Figure S5.** The effects of incubation time on Aβ promoted tau aggregation. **Figure S6.** Aβ oligomers do not affect α-synuclein seeding. **Figure S7.** Western blot analysis of SH-SY5Y cell lysates treated with Aβ oligomers and tau seeds. **Figure S8.** ELISA quantification of tau concentrations in SH-SY5Y cell lysates. **Figure S9.** Aβ oligomers promote the internalization of tau seeds in wild-type mice primary hippocampal neurons. **Figure S10.** Fluorescence-microscopy images of tau biosensor cells seeded with tau fibrils 24 h after pretreatment with Aβ oligomers at 100, 200 and 500 nM. **Figure S11.** Dose-response analysis of the effects of different Aβ species on tau seeding in tau biosensor cells. **Figure S12.** Characterization of full-length tau 40 self-assembly. **Figure S13.** EM image of sonicated tau RD. **Figure S14.** Pretreatment of Aβ oligomers promotes tau seeding in the presence of lipofectamine. **Figure S15.** Statistical tests of normal distribution and equal variability of the data.

## Data Availability

Online Content Methods, along with any additional Extended Data display items and Source Data, are available in the online version of the paper; references unique to these sections appear only in the online paper.

## Abbreviations

AD: Alzheimer’s disease
Aβ: Amyloid β-protein
CFP: Cyan-fluorescent protein
FRET: Fluorescence resonance energy transfer
GFP: Green-fluorescent protein
RD: Repeat domain
YFP: Yellow-fluorescent protein
