# Correction to: Amyloid β-protein oligomers promote the uptake of tau fibril seeds potentiating intracellular tau aggregation

**DOI:** 10.1186/s13195-021-00824-5

**Published:** 2021-04-19

**Authors:** Woo Shik Shin, Jing Di, Qin Cao, Binsen Li, Paul M. Seidler, Kevin A. Murray, Gal Bitan, Lin Jiang

**Affiliations:** 1 Department of Neurology, David Geffen School of Medicine, UCLA, Los Angeles, CA 90095 USA; 2 Departments of Chemistry and Biochemistry and Biological Chemistry, UCLA-DOE Institute, UCLA, Los Angeles, CA 90095-1570 USA; 3 Brain Research Institute, and Molecular Biology Institute, UCLA, Los Angeles, CA 90095 USA

**Correction to: Alz Res Therapy 11, 86 (2019)**

**https://doi.org/10.1186/s13195-019-0541-9**

Following publication of the original article [1], the authors reported an error in the supplementary Fig. 4. One of the fluorescent cell images from control “No Aβ” experiments was misplaced. The corrected supplementary Fig. 4 is given here as Fig. 1.

**Fig. 1:**
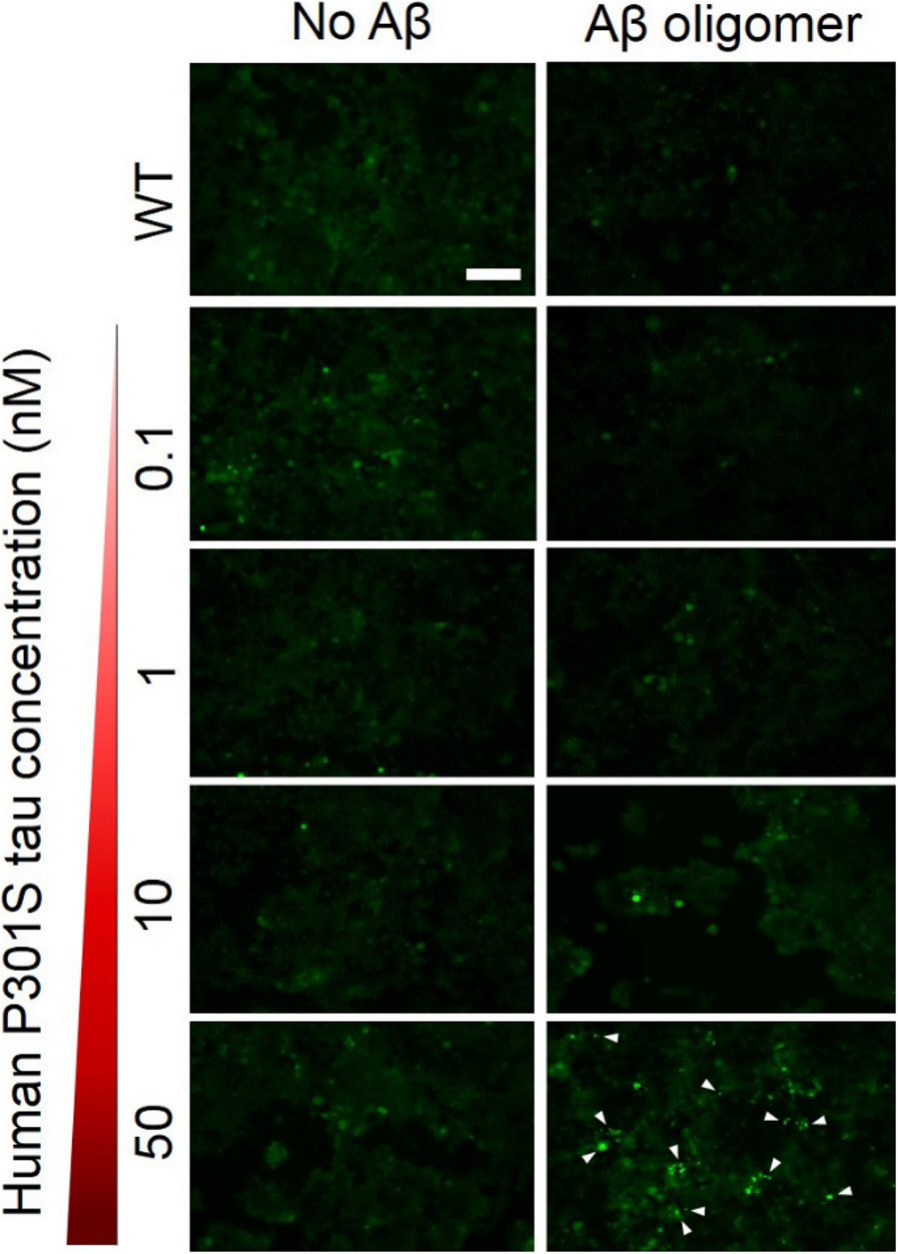
Aβ oligomer pretreatment promotes intracellular tau aggregation when biosensor cells are seeded with brain extracts from transgenic mice expressing human P301S-tau
