# Supplementary material for: Amyloid β-protein oligomers promote the uptake of tau fibril seeds potentiating intracellular tau aggregation
Source: Alzheimers Res Ther. 2019 Oct 18;11:86. doi: 10.1186/s13195-019-0541-9 (PMC6800506; doi:10.1186/s13195-019-0541-9)
Supplement: Supplementary file 1 — Additional file 1: Figure S1. Characterization of Aβ (1–42) preparations. Figure S2. The effect of different Aβ species on tau aggregation in the absence of tau seeds in biosensor cells. Figure S3. When treated Aβ oligomers up to two weeks in the absent of tau seeds, biosensor cells display negligible tau aggregation. Figure S4. Aβ oligomer pretreatment promotes intracellular tau aggregation when biosensor cells are seeded with brain extracts from transgenic mice expressing human P301S-tau. Figure S5. The effects of incubation time on Aβ promoted tau aggregation. Figure S6. Aβ oligomers do not affect α-synuclein seeding. Figure S7. Western blot analysis of SH-SY5Y cell lysates treated with Aβ oligomers and tau seeds. Figure S8. ELISA quantification of tau concentrations in SH-SY5Y cell lysates. Figure S9. Aβ oligomers promote the internalization of tau seeds in wild-type mice primary hippocampal neurons. Figure S10. Fluorescence-microscopy images of tau biosensor cells seeded with tau fibrils 24 h after pretreatment with Aβ oligomers at 100, 200 and 500 nM. Figure S11. Dose-response analysis of the effects of different Aβ species on tau seeding in tau biosensor cells. Figure S12. Characterization of full-length tau 40 self-assembly. Figure S13. EM image of sonicated tau RD. Figure S14. Pretreatment of Aβ oligomers promotes tau seeding in the presence of lipofectamine. Figure S15. Statistical tests of normal distribution and equal variability of the data. [file 13195_2019_541_MOESM1_ESM.docx]

**Supplementary Figures for**

**Amyloid β-protein oligomers promote the uptake of tau fibril seeds potentiating intracellular tau aggregation**

Woo Shik Shin^1^, Jing Di^1^, Qin Cao^2^, Binsen Li^1^, Paul M. Seidler^2^, Kevin A. Murray^2^, Gal Bitan^1,3^, Lin Jiang^1,3 *^

1 Department of Neurology, David Geffen School of Medicine, UCLA, Los Angeles CA 90095;

2 Departments of Chemistry and Biochemistry and Biological Chemistry, UCLA-DOE Institute, UCLA, Los Angeles CA 90095-1570

3 Brain Research Institute, and Molecular Biology Institute, UCLA, Los Angeles CA 90095;

**Supplemental Figure S1.** **Characterization of Aβ(1-42) preparations.**

**Supplemental Figure S2. The effect of different Aβ species on tau aggregation in the absence of tau seeds in biosensor cells.**

**Supplemental Figure S3. When treated Aβ oligomers up to two weeks in the absent of tau seeds, biosensor cells display negligible tau aggregation.**

**Supplemental Figure S4. Aβ oligomer pretreatment promotes intracellular tau aggregation when biosensor cells are seeded with brain extracts from transgenic mice expressing human P301S-tau.**

**Supplemental Figure S5.** **The effects of incubation time on Aβ promoted tau aggregation.**

**Supplemental Figure S6. Aβ oligomers do not affect α-synuclein seeding.**

**Supplemental Figure S7. Western blot analysis of SH-SY5Y cell lysates treated with Aβ oligomers and tau seeds.**

**Supplemental Figure S8. ELISA quantification of tau concentrations in SH-SY5Y cell lysates.**

**Supplemental Figure S9. Aβ oligomers promote the internalization of tau seeds** **in wild-type mice primary hippocampal neurons.**

**Supplemental Figure S10. Fluorescence-microscopy images of tau biosensor cells seeded with tau fibrils 24 h after pretreatment with Aβ oligomers at 100, 200 and 500nM**.

**Supplemental Figure S11.** **Dose-response analysis of the effects of different Aβ species on tau seeding in tau biosensor cells.**

**Supplemental Figure S12. Characterization of full-length tau 40 self-assembly.**

**Supplemental Figure S13. EM image of sonicated tau RD.**

**Supplemental Figure S14. Pretreatment of Aβ oligomers promotes tau seeding in the presence of lipofectamine. Supplemental Figure S15. Statistical tests of normal distribution and equal variability of the data.**


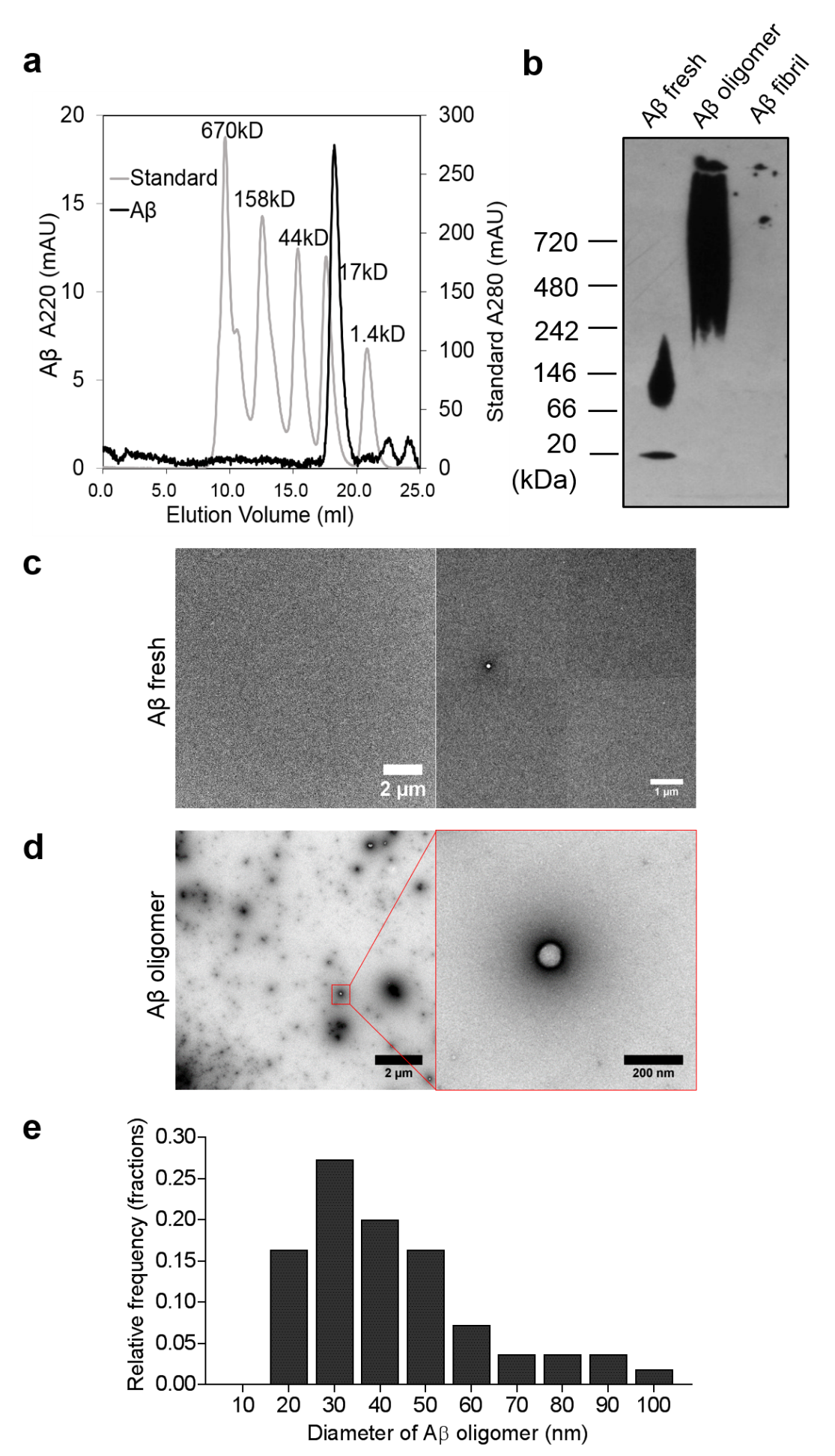


**Supplemental Figure S1.** **Characterization of Aβ(1-42) preparations.** **a.** Purified Aβ shows a single peak in SEC fractionation. **b**. Native PAGE/western blot analysis of freshly prepared, oligomeric, and fibrillar Aβ, fractionated on a 12% Bis-Tris Native gel and probed with monoclonal antibody 6E10. **c.** EM images of freshly prepared Aβ sample. The majority of the images did not show aggregated species (left panel). Oligomers were seen occasionally (right panel). **d.** EM images of spherical Aβ oligomers. **e.** Diameter distribution of Aβ oligomers (nm).


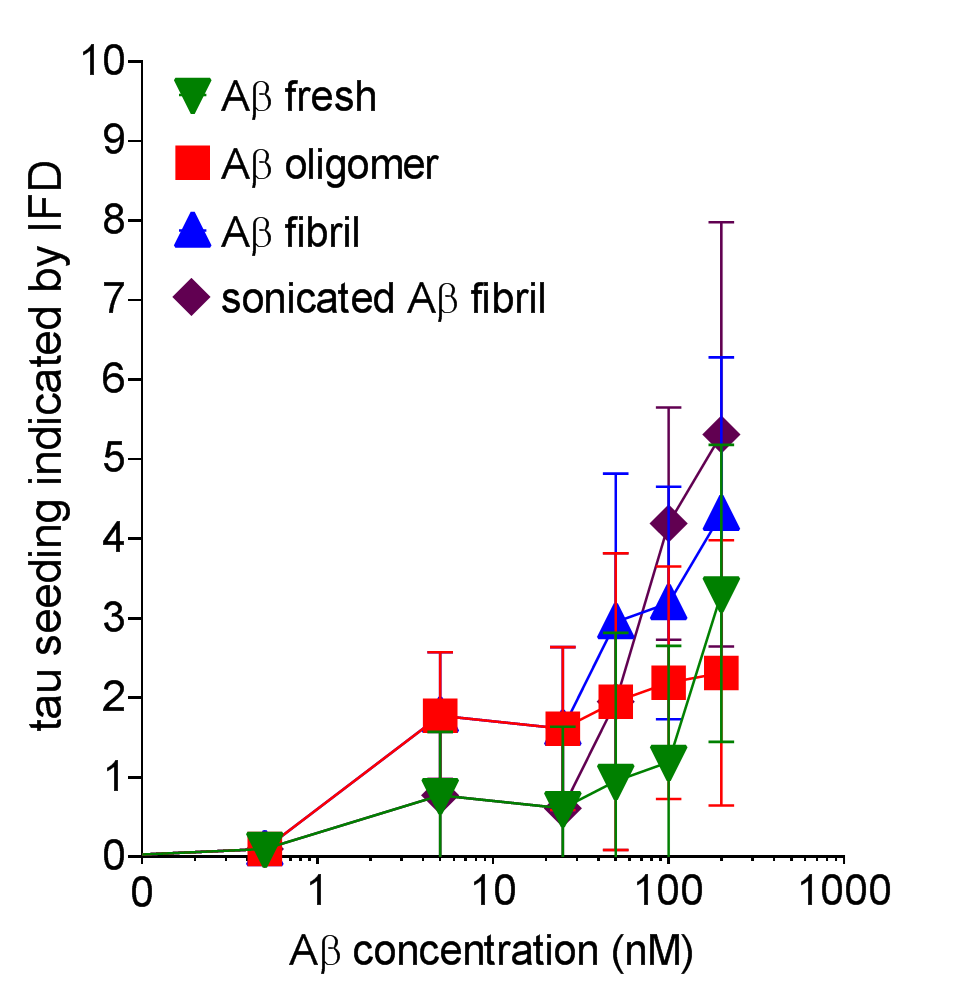


**Supplemental Figure S2. The effect of different Aβ species on tau aggregation in the absence of tau seeds in biosensor cells.** Various concentrations of freshly prepared, oligomeric, non-sonicated fibrils, or sonicated Aβ fibrils were added to the biosensor cells in the absence of tau seeds. After 48 hours, intracellular tau aggregation was measured by flow cytometry and quantified as Integrated FRET Density (IFD). The data are presented as mean ± SD.


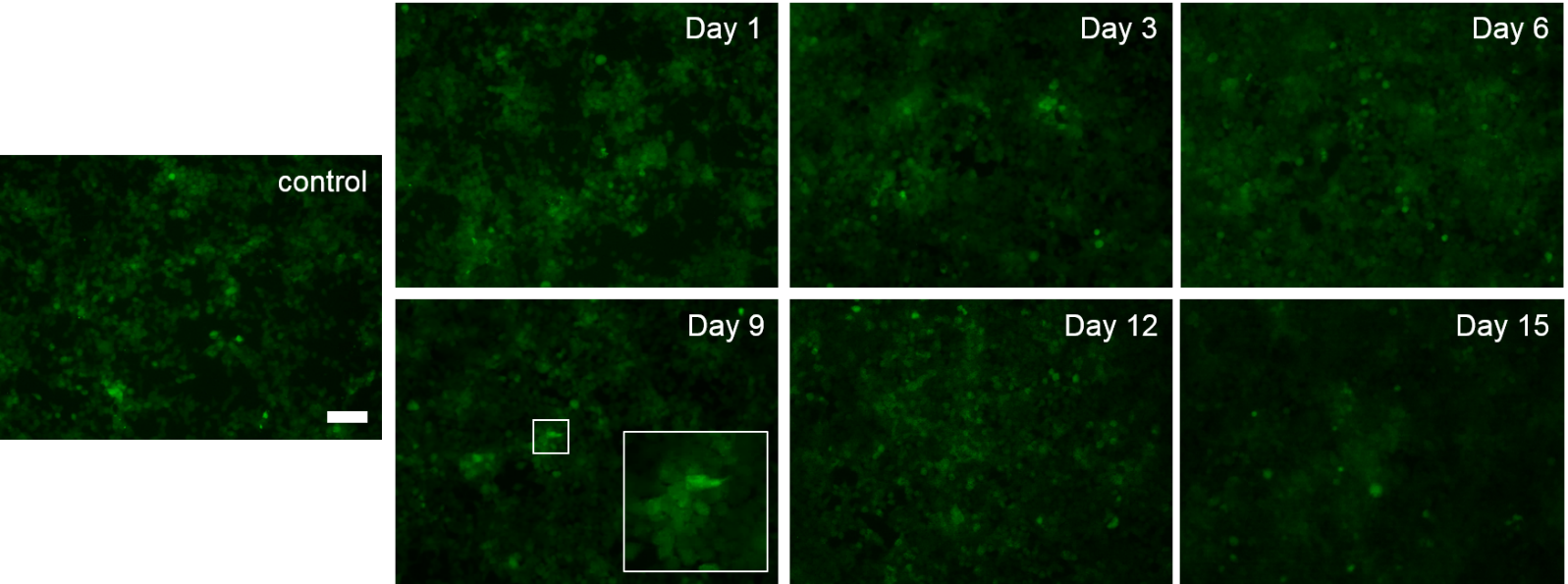


**Supplemental Figure S3. When treated Aβ oligomers up to two weeks in the absent of tau seeds, biosensor cells display negligible tau aggregation.** Represenative fluoresence-microscopy images are shown of biosenser cells at different time points with replenishment of 200 nM Aβ oligomers every three days for two weeks. Bright fluorsecent areas (exemplified in a Day 9 image inset) are cells with high levels of diffuse background fluorescence, not aggregated tau. The scale bar denotes 50 μm.


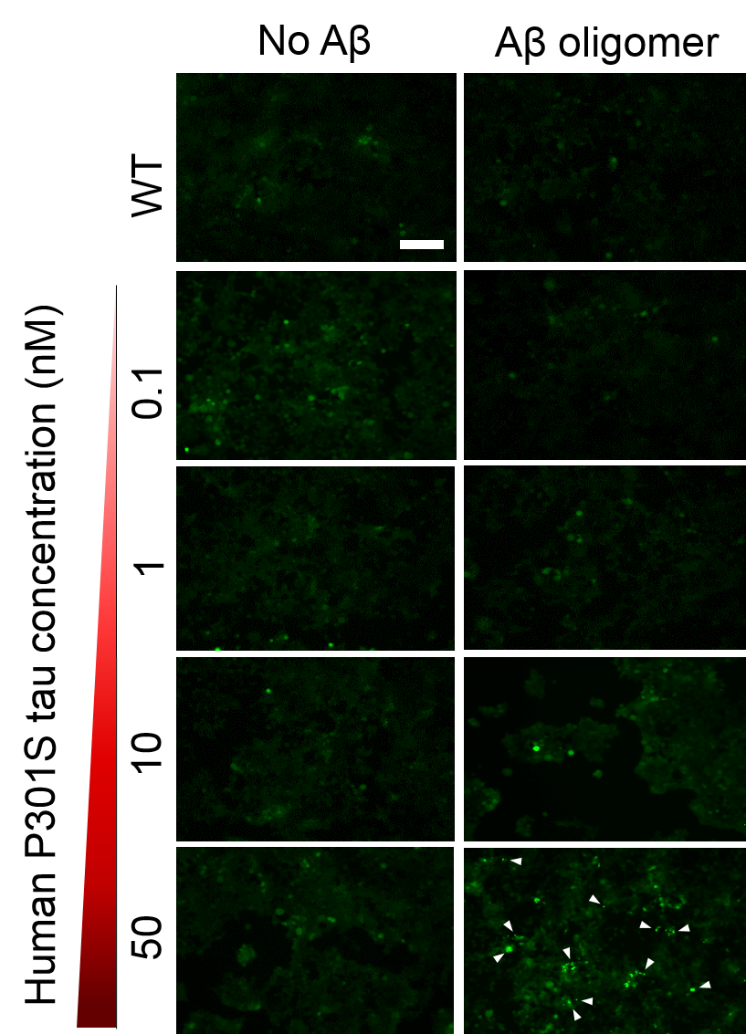


**Supplemental Figure S4. Aβ oligomer pretreatment promotes intracellular tau aggregation when biosensor cells are seeded with brain extracts from transgenic mice expressing human P301S-tau.** Fluorescence-microscopy images of tau biosensor cells seeded with brain extracts from mice expressing human P301S tau in the absence (left panels) or presence of pretreatment with 500 nM Aβ oligomers (right panels). ELISA was used to quantify human tau expressed in mouse brain extracts. The scale bar denotes 50 μm.


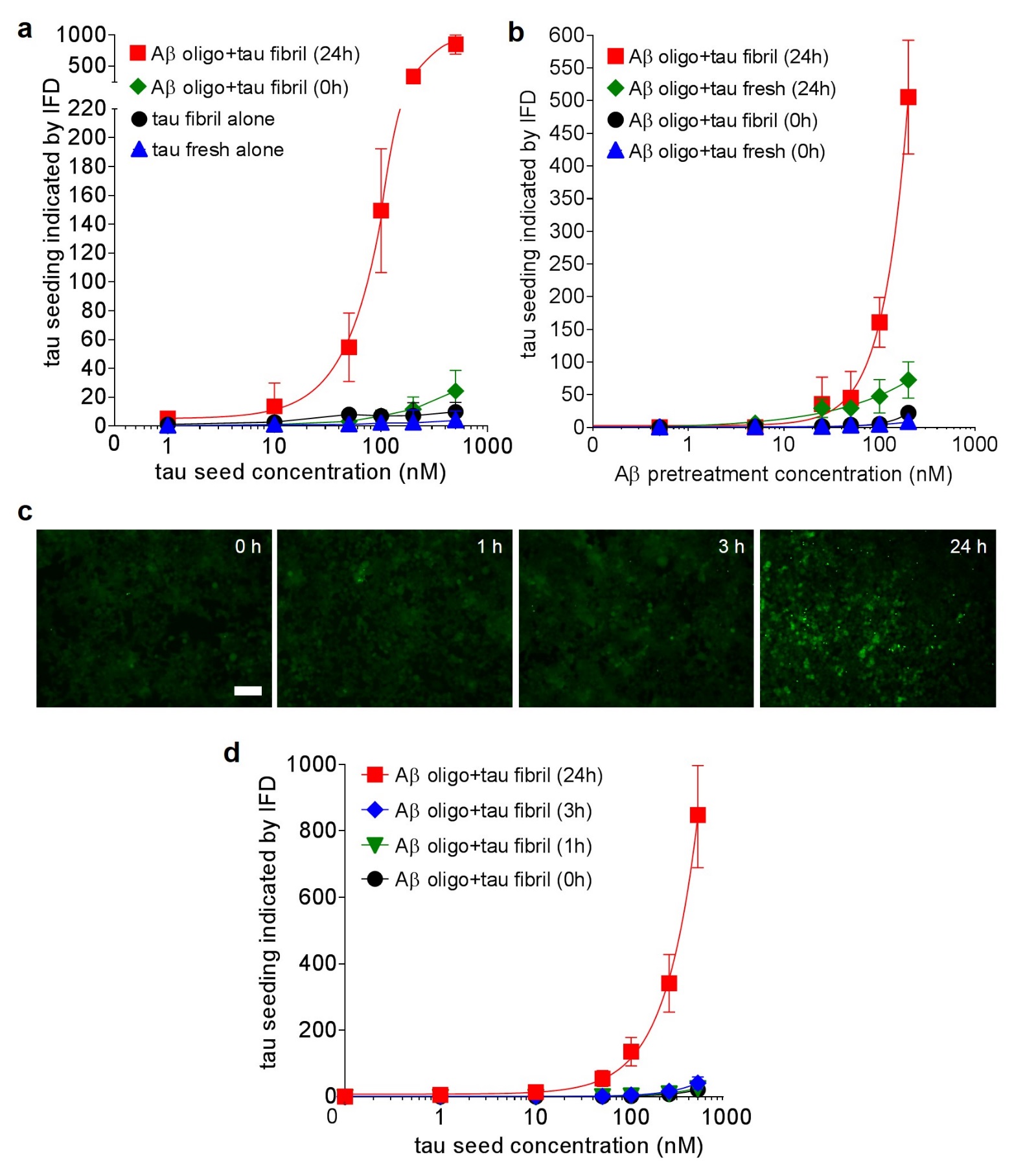


**Supplemental Figure S5.** **The effects of incubation time on Aβ promoted tau aggregation.** **a.** Tau biosensor cells treated with various concentrations of freshly prepared tau (blue) and tau fibril (black) alone. The simultaneous addition of Aβ oligomer at the same time as the tau fibril seed (0 h, green) yielded intracellular tau aggregation similar to that of tau fibril seed alone, which are both much lower compared to cells treated with tau seed 24 hours after Aβ oligomer pretreatment (24 h, red). **b.** The simultaneous treatment of tau seeds and various concentrations of Aβ oligomer (0.5 to 200 nM) did not result in a significant promotion of tau seeding (green) when compared to cells treated with tau seed 24 hours after Aβ oligomer pretreatment (red). Tau seeding was quantified using integrated FRET density (IFD) by flow cytometry. **c.** Fluorescence-microscopy images of tau biosensor cells seeded with tau fibrils 0 h, 1 h, 3 h, and 24 h after pretreatment with 200 nM Aβ oligomer. The scale bar denotes 50 μm. **d.** The different time point of Aβ oligomer incubation before tau seed addition (0, 1, 3, 24 h).

**
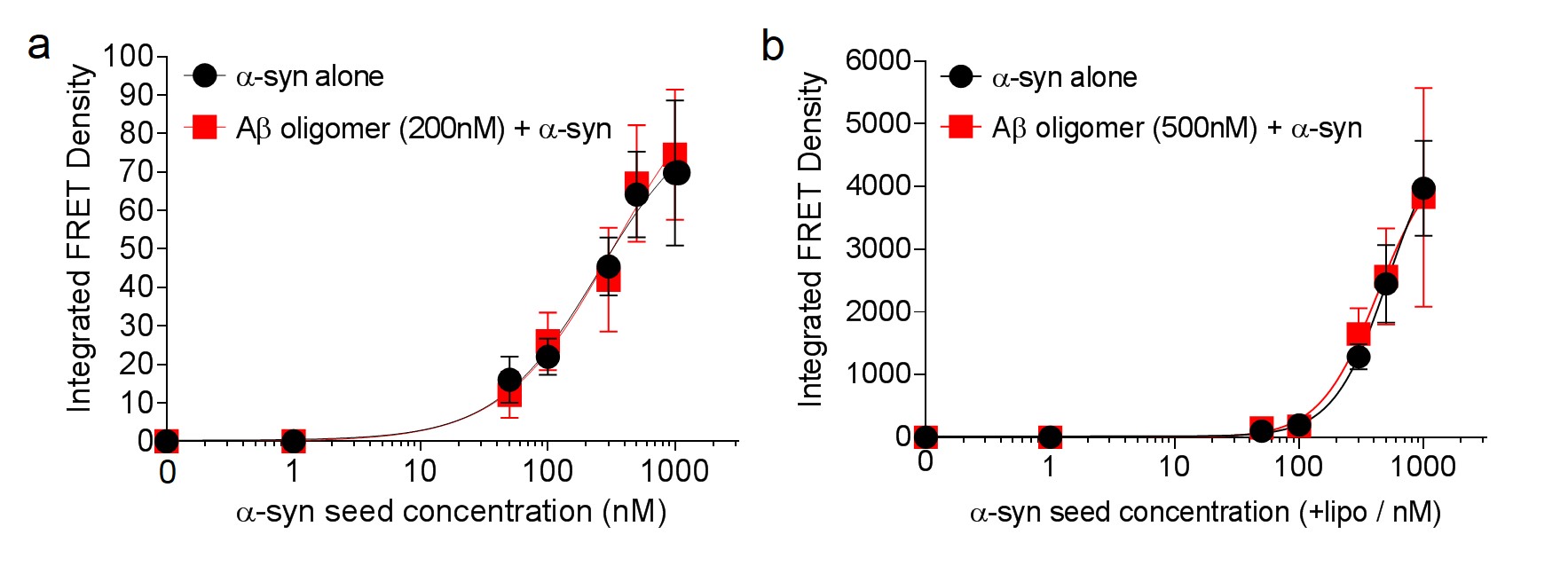
**

**Supplemental Figure S6. Aβ oligomers do not affect α-synuclein seeding.** Sonicated α-synuclein fibrils at concentrations ranging from 1 nM to 1000 nM were added to α-synuclein biosensor cells in the absence or presence of Aβ oligomer pretreatment. **a.** After 24 h pretreatment with 200 nM Aβ oligomers, α-synuclein fibril seeds were added to cells without lipofectamine, a cell transfection reagent. Aggregation of endogenous α-synuclein was measured by integrated FRET density. The data are presented as mean ± SD (n = 6). **b.** After 24 h pretreatment with 500 nM Aβ oligomers, α-synuclein seeds were added to cells in the presence of lipofectamine.

**
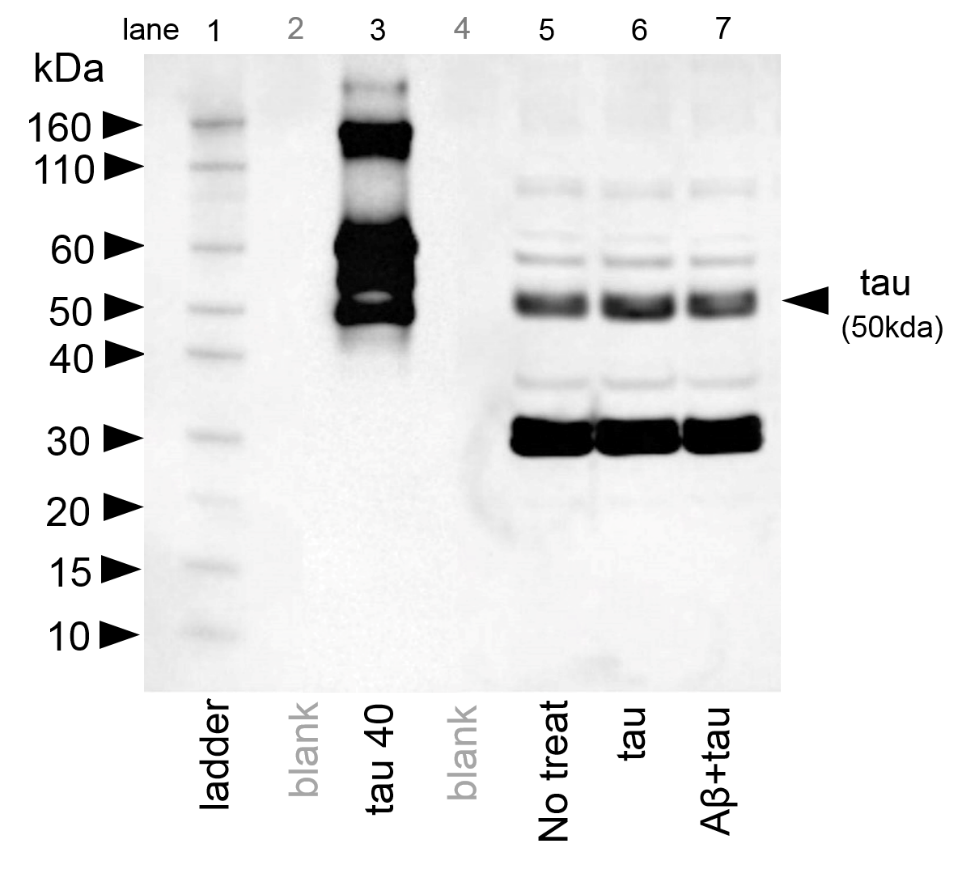
**

**Supplemental Figure S7. Western blot analysis of SH-SY5Y cell lysates treated with Aβ oligomers and tau seeds.** The tau in SH-SY5Y cell lysates were analyzed by western blot using monoclonal antibody HT7. SeeBlue Plus2 Pre-stained protein ladder (Thermo Fisher Scientific) were used as standard. Recombinant tau 40 was used as a positive control (lane 3). Lysates from non-treated cells were used as a negative control (lane 5). Cells treated with tau seed (lane 6) and cells with pretreatment of Aβ oligomer and tau seed (lane 7) were analyzed.

**
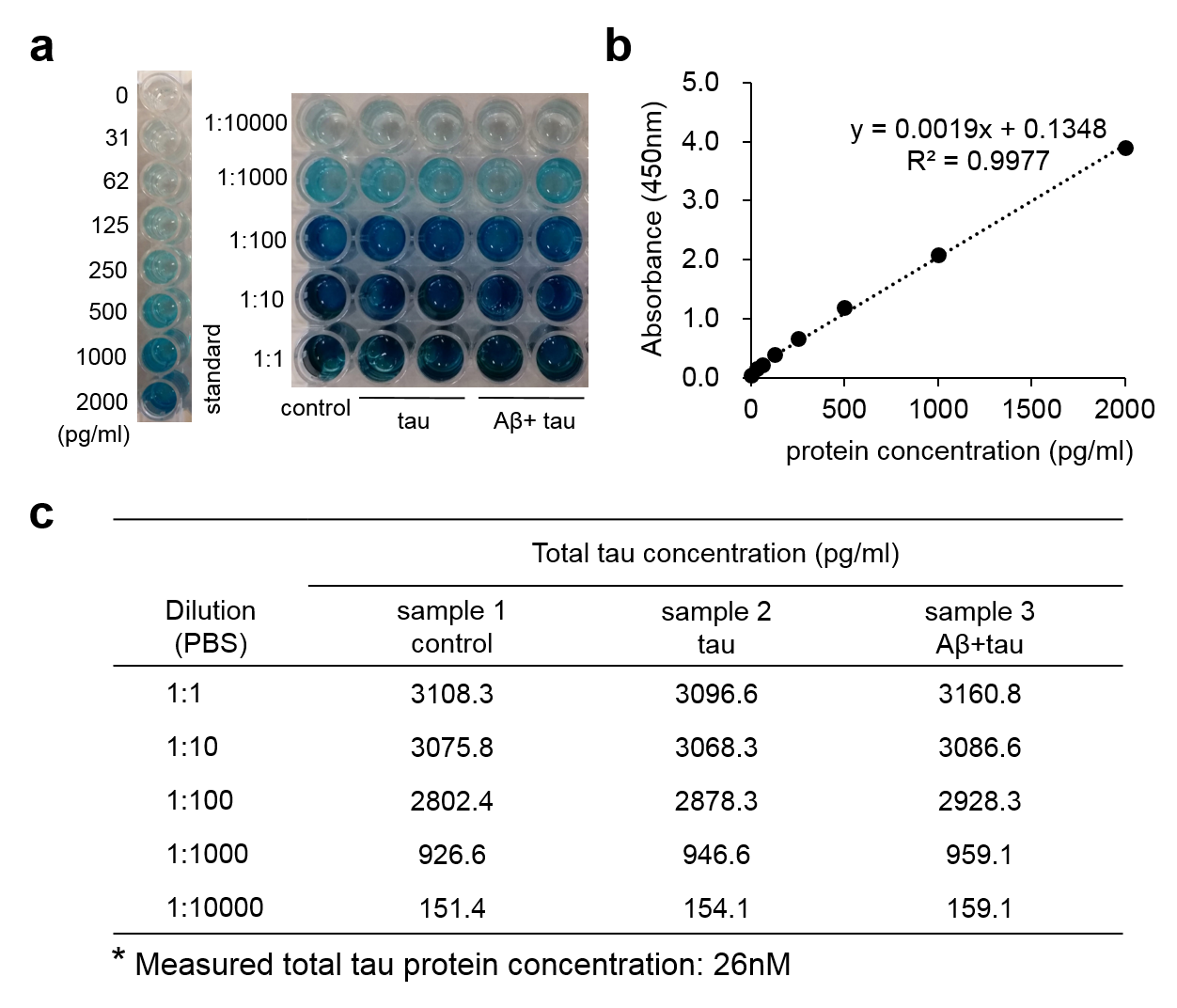
**

**Supplemental Figure S8. ELISA quantification of tau concentrations in SH-SY5Y cell lysates.** **a.** Serial dilutions of cell lysates ranging from 1:1 to 1:10,000, and dilutions of protein standard are shown (left side). **b.** The absorbance reading of protein standards with a series of dilutions. **c.** The total tau concentration of each diluted cell lysate supernatant was calculated based on the standard dilution curve. The cell lysate supernatants treated with tau seeds alone or with Aβ pretreatment had very similar tau concentration, and the final tau concentration was estimated as 26 nM.


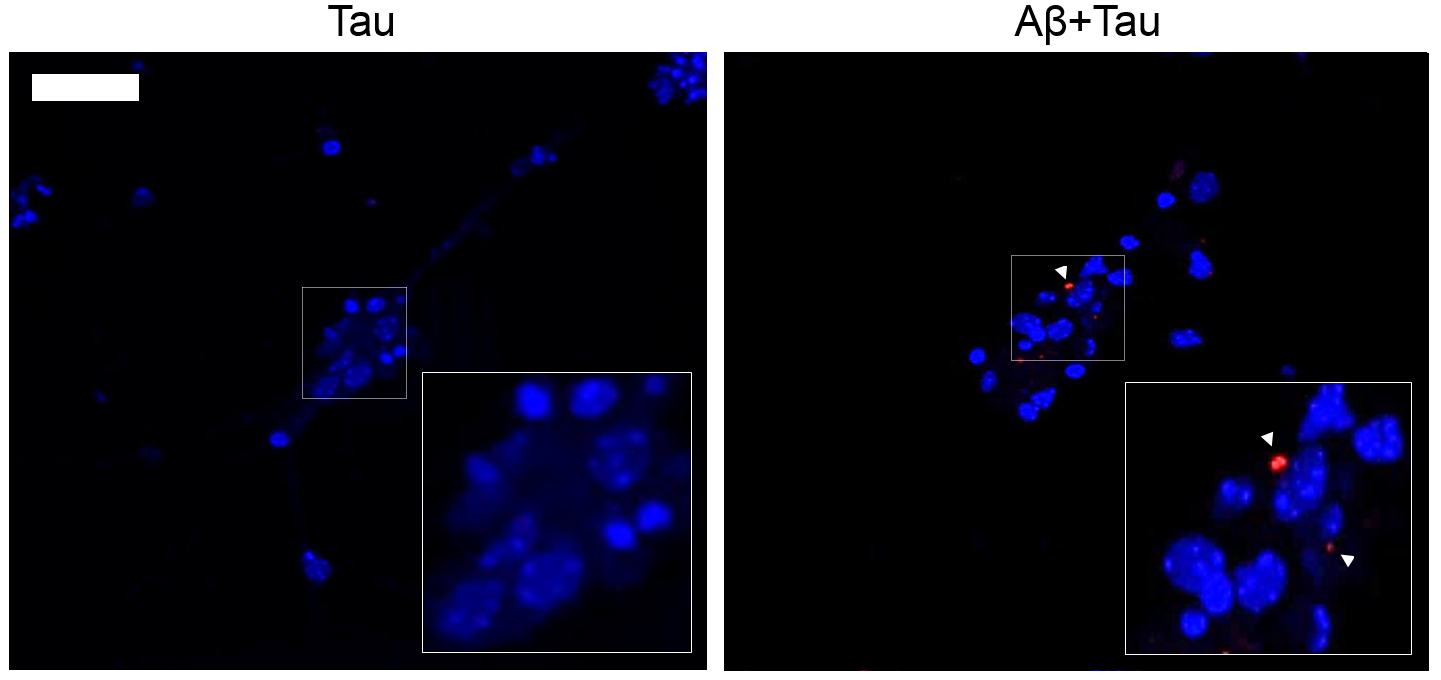


**Supplemental Figure S9. Aβ oligomers promote the internalization of tau seeds** **in wild-type mice primary hippocampal neurons.** Primary hippocampal neurons from wild-type mice were pretreated with 200 nM Aβ oligomers at 24 h and 500 nM tau seeds at 48 h. At 72 h, after three PBS washes, immunostaining was performed with DAPI (blue) and human tau antibody HT7 (red). White triangles indicate internalized tau seeds. The scale bar denotes 20 μm.


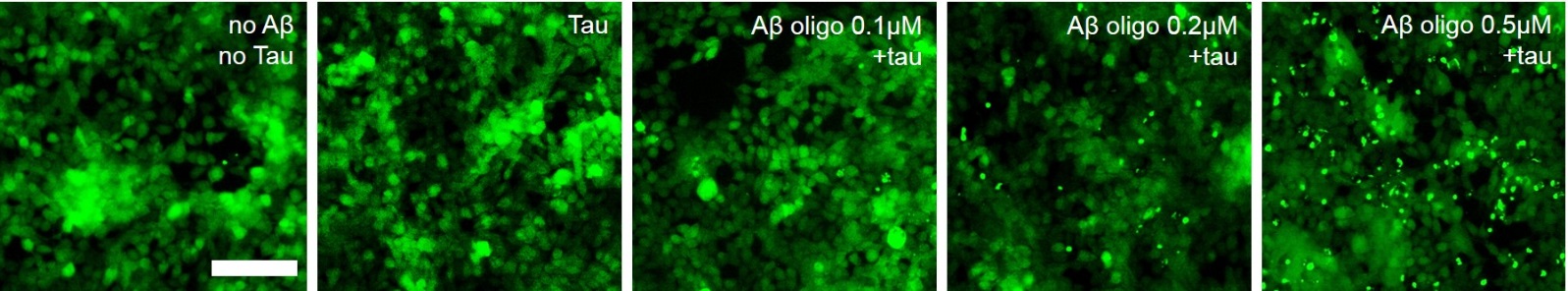


**Supplemental Figure S10. Fluorescence-microscopy images of tau biosensor cells seeded with tau fibrils 24 h after pretreatment with Aβ oligomers at 100, 200 and 500nM**. Tau aggregates are indicated by green puncta.The scale bar denotes 50 μm.


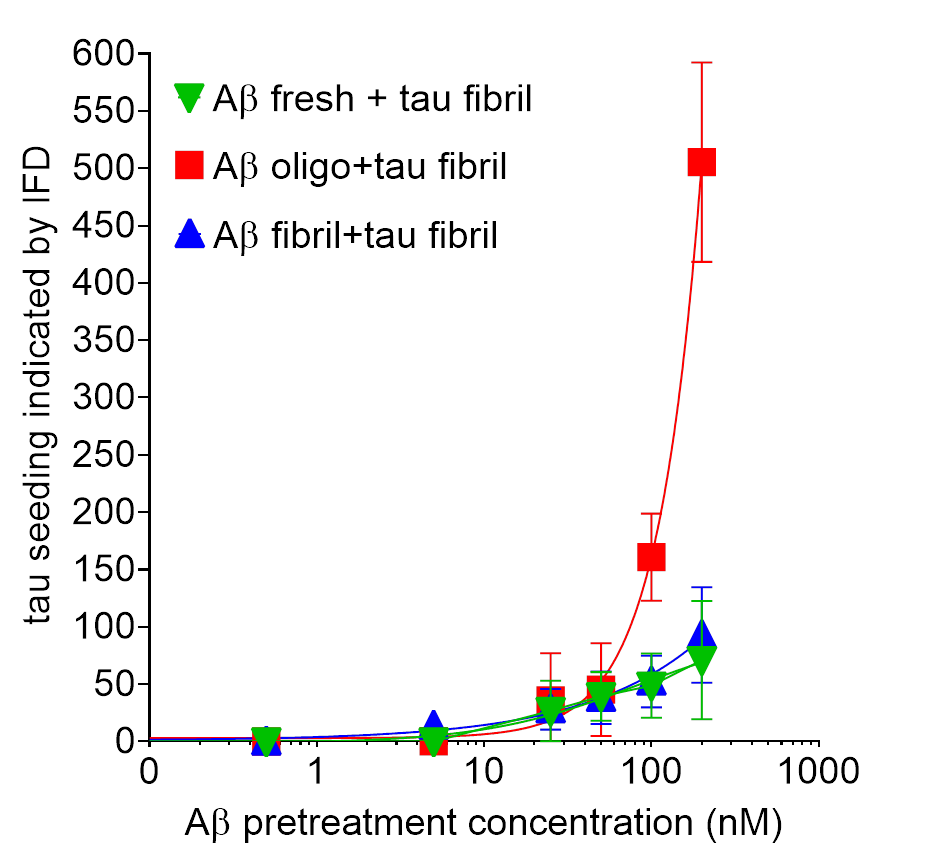


**Supplemental Figure S11.** **Dose-response analysis of the effects of different Aβ species on tau seeding in tau biosensor cells.** Pretreatment with increasing concentrations of freshly prepared Aβ, Aβ oligomers, or fibrils, from 0.5 to 200 nM, followed by 200 nM tau RD seeds leads to increased intracellular tau aggregation. Treatment with Aβ oligomer at concentrations higher than 200 nM leads to non-negligible cell death, therefore, data are not shown.


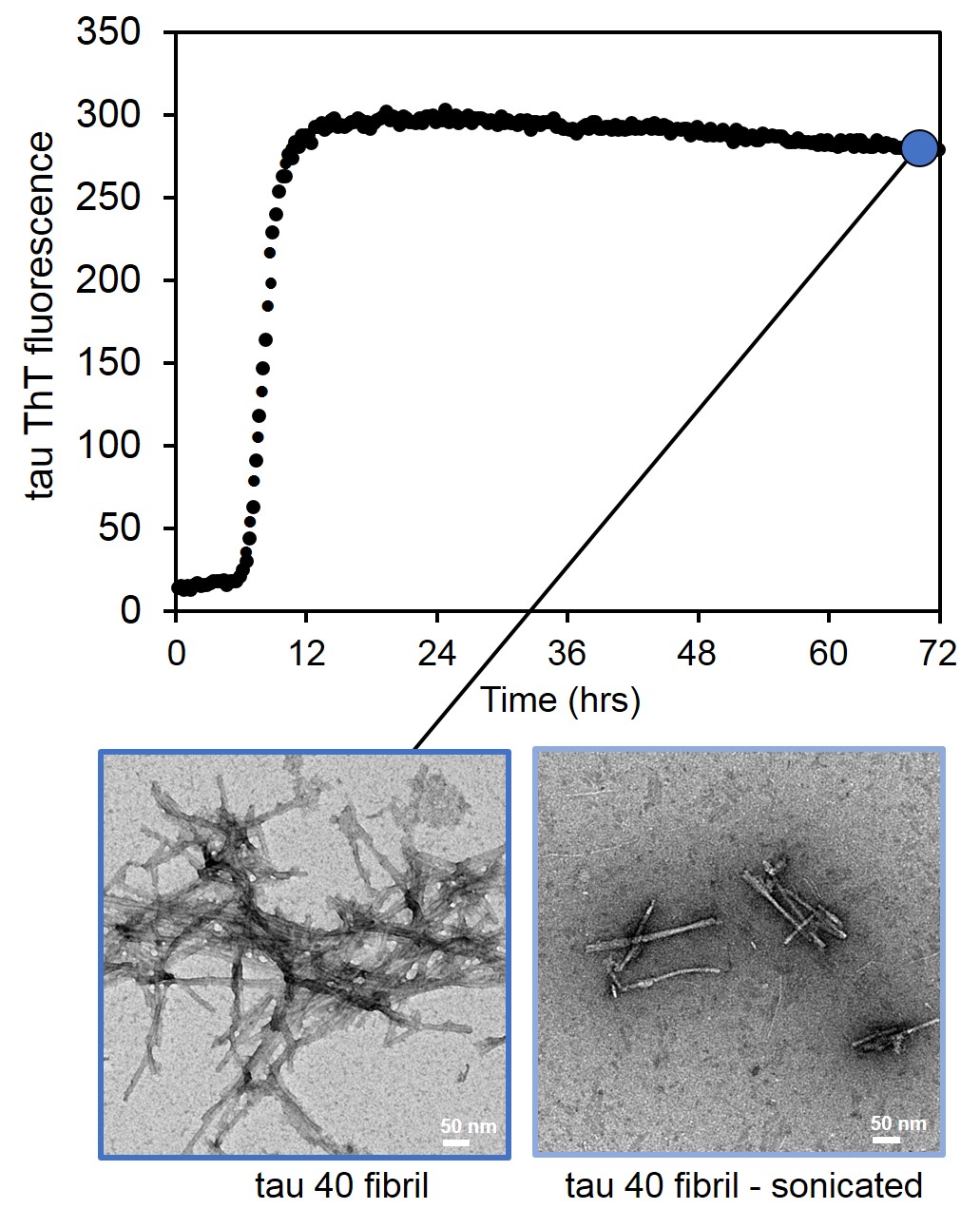


**Supplemental Figure S12. Characterization of full-length tau 40 self-assembly.** ThT fluorescence (upper panel) and EM images (lower panel) of full-length tau 40 fibrils before (left) and after (right) sonication. EM images were taken of the sample after 72 hours incubation with shaking at 37^o^C.


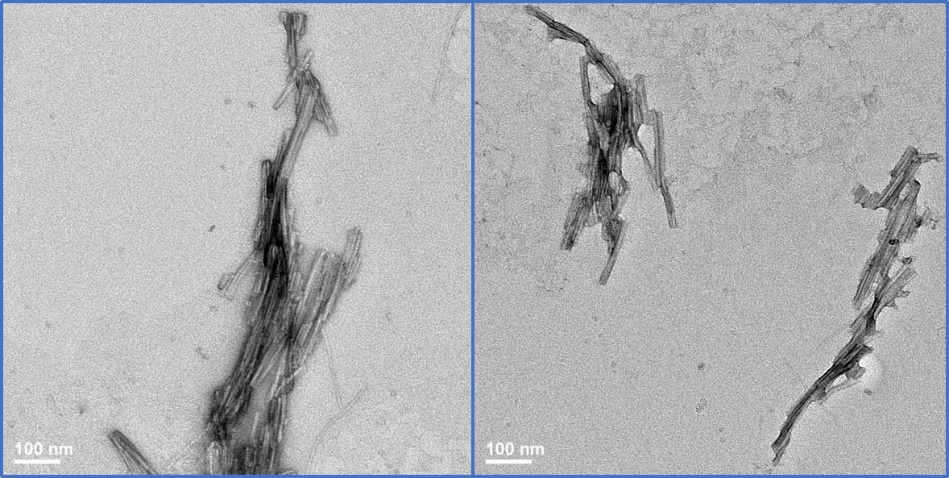


**Supplemental Figure S13. EM image of sonicated tau RD.** Tau RD fibrils used for seeding experiments were diluted with Opti-MEM and sonicated for 10 min in an ultrasonic water bath and then imaged by EM.


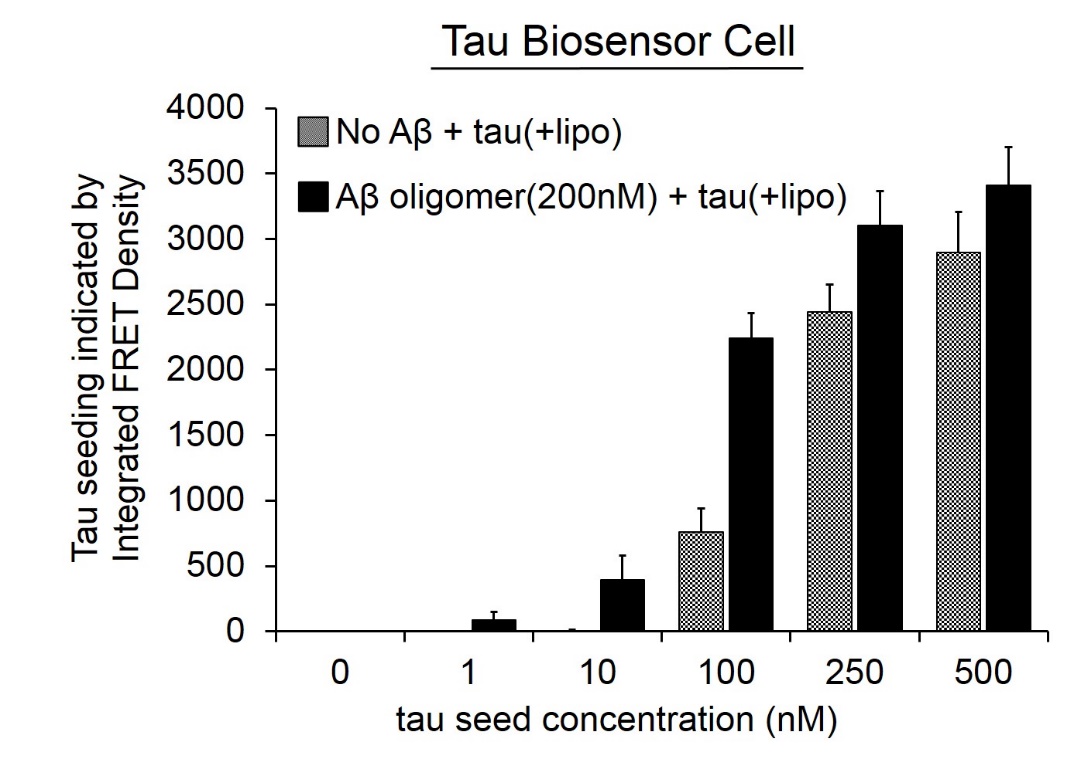


**Supplemental Figure S14. Pretreatment of Aβ oligomers promotes tau seeding in the presence of lipofectamine.** Flow-cytometry-based FRET quantification of intracellular tau aggregation of tau biosensor cells seeded by recombinant tau RD fibrils. The enhancement of tau aggregation by Aβ oligomers pretreatment occurs in a dose-dependent manner in the presence of cell transfection reagent, lipofectamine.


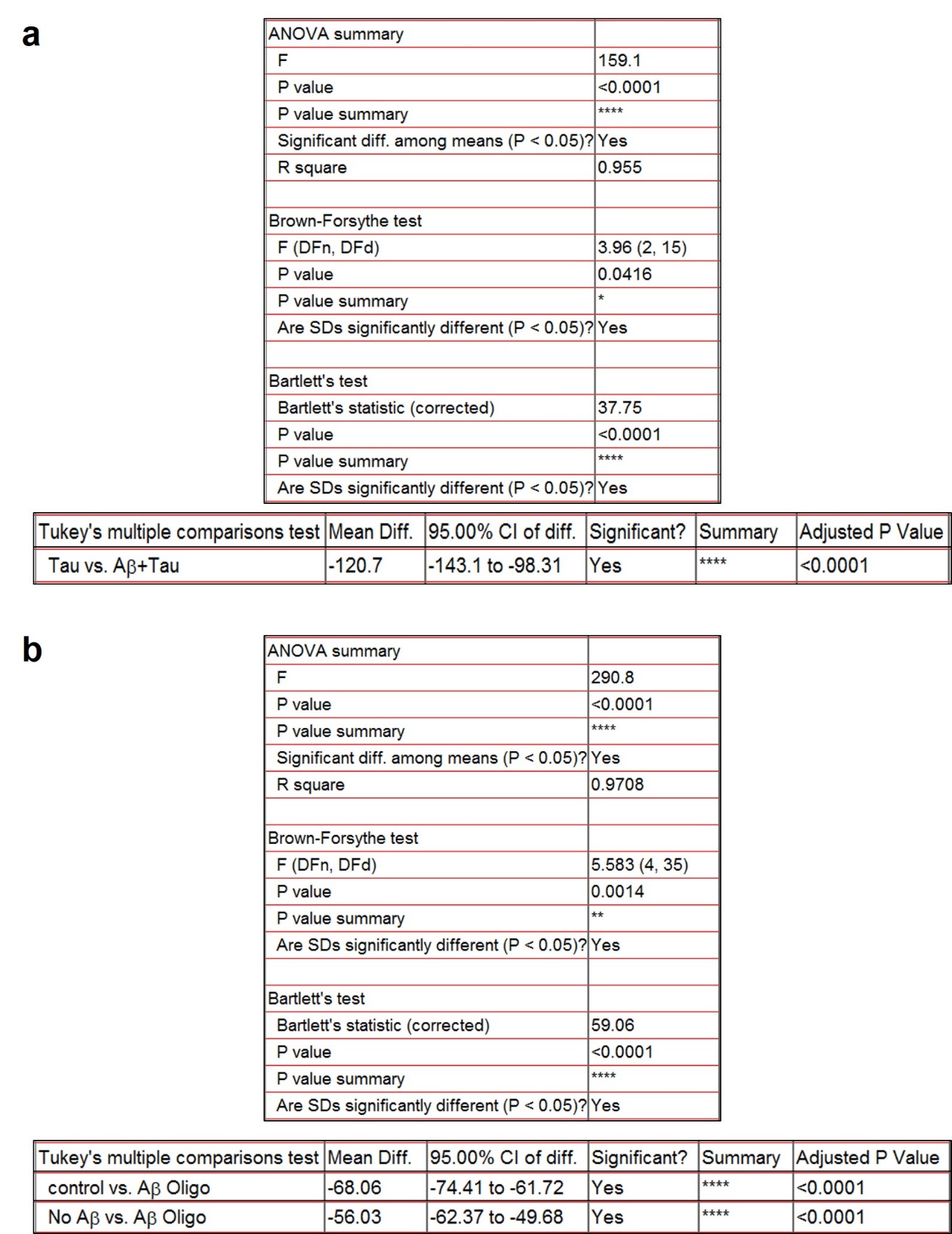


**Supplemental Figure S15. Statistical tests of normal distribution and equal variability of the data.** The results (**a.** for Figure 3c **b.** for Figure 4b) from the Bartlett's test and Brown-Forsythe test to support the use of the parametric statistical tests. The test were performed using GraphPad Prism software ver. 7.0.
